# Development of behavioural profile in the Northern common boa (*Boa imperator*): Repeatable independent traits or personality?

**DOI:** 10.1371/journal.pone.0177911

**Published:** 2017-05-24

**Authors:** Olga Šimková, Petra Frýdlová, Barbora Žampachová, Daniel Frynta, Eva Landová

**Affiliations:** 1 Department of Zoology, Faculty of Science, Charles University, Prague, Czech Republic; 2 National Institute of Mental Health, Klecany, Czech Republic; University of Pretoria, SOUTH AFRICA

## Abstract

Recent studies of animal personality have focused on its proximate causation and ecological and evolutionary significance in particular, but the question of its development was largely overlooked. The attributes of personality are defined as between-individual differences in behaviour, which are consistent over time (differential consistency) and contexts (contextual generality) and both can be affected by development. We assessed several candidates for personality variables measured in various tests with different contexts over several life-stages (juveniles, older juveniles, subadults and adults) in the Northern common boa. Variables describing foraging/feeding decision and some of the defensive behaviours expressed as individual average values are highly repeatable and consistent. We found two main personality axes—one associated with foraging/feeding and the speed of decision, the other reflecting agonistic behaviour. Intensity of behaviour in the feeding context changes during development, but the level of agonistic behaviour remains the same. The juveniles and adults have a similar personality structure, but there is a period of structural change of behaviour during the second year of life (subadults). These results require a new theoretical model to explain the selection pressures resulting in this developmental pattern of personality. We also studied the proximate factors and their relationship to behavioural characteristics. Physiological parameters (heart and breath rate stress response) measured in adults clustered with variables concerning the agonistic behavioural profile, while no relationship between the juvenile/adult body size and personality concerning feeding/foraging and the agonistic behavioural profile was found. Our study suggests that it is important for studies of personality development to focus on both the structural and differential consistency, because even though behaviour is differentially consistent, the structure can change.

## Introduction

The study of animal personality has recently become a central topic of evolutionary behavioural ecology (reviewed in [1–3]). Attributes of personality, also referred to as a temperament or coping style [4], are defined as between-individual differences in behaviour consistent both over time and contexts [5–7]. According to [3], a context is broadly defined as: “all of external stimuli surrounding an individual when it expresses a given behaviour”, including any stimuli interacting with the animal [8–15] and situation sensu Sih and his colleagues [6], are currently incorporated in one term. On the other hand, a behavioural syndrome is a term for a series of correlated behaviours, which reflects the between-individual consistency in behaviours across two or more situations [6]. Personality can be found both in vertebrates [5] and invertebrates [16, 17], however, the number of studies dealing with personality of warm-blooded tetrapods (birds: [18–22] and mammals: [23–26]) highly exceeds the number of those focused on the cold-blooded ones (amphibians: [27–29]; reptiles: [8, 9, 30–32]). Recently, personality development over ontogeny is the key question of personality studies describing theoretical models as well as their experimental testing [3, 33–36].

Stamps and Groothuis [3] aimed to summarize definitions of key terms used in the study of animal personality development. Differential consistency (according to [3, 37]) describes how inter-individual behavioural differences in a single context are maintained over time. We conceptualize these as repeatability, an important tool in personality studies and one of the requirements for assessment of personality traits [4], allowing us to compare inter- and intra-individual variability [38, 39]. Differential consistency (repeatability) thus reflects how individuals vary in a particular behaviour at two or multiple points in time, e.g., if they are more/moderately/less explorative in relation to others. In an extensive meta-analysis of various behavioural tests Bell and her colleagues [2] showed that mean repeatability is about 0.37. We can expect this value (or even higher) in variables describing various aspects of animal personality. However, repeatability in ectotherms is reported to be slightly lower than in endotherms (0.24), at least in laboratory conditions [2].

The second feature of animal personality is the context generality, which is usually measured as a correlation of different behaviours (from different behavioural tests) or similar behaviours (e.g., the level of aggressive or defensive behaviour) in different contexts (e.g., meeting familiar or new intruders, in the home or neutral arena, etc.). Correlations between behaviours or various contexts that are stable over time are called structural consistency [3].

The current concept assumes equal importance of contextual and temporal axes of personality [3]. Nevertheless, studies dealing with personality development usually lack comparisons in several contexts (but see [18, 40–43]). Structural consistency is usually studied through aspects of boldness [28, 35, 40, 41, 44–48] and/or exploratory behaviour [18, 36, 49]. Sometimes, agonistic behaviour towards a conspecific and its correlation with behaviour in other contexts is studied as well [40, 44, 48]. Agonistic behaviour and boldness can also be correlated with various physiological stress markers, such as corticosterone levels or the heart rate response to stress, which is larger in more aggressive animals [32, 50–52]. The structural consistency is not studied so often in comparison with the differential one. Moreover, long-term studies of personality covering all important life-stages (new-borns, juveniles, subadults and adults) with important life-history ontogenetic transitions are almost absent. Recent studies revealed mixed records of personality consistency throughout the ontogeny. A surprising level of consistency including metamorphosis has been found in some invertebrates [45, 46] as well as in lake frogs [28]. Partial inconsistency has been discovered in sticklebacks [40], yellow-belied marmots [47], and dumpling squids [41]. Moreover, structural and differential consistency are usually studied with a shorter inter-test interval without considering the ontogenetic change [2, 3, 53].

Behavioural consistency in time as an important assumption of personality is complicated by the inevitable animal’s ontogeny [37, 44, 54, 55]. The growth and maturation are inherently connected with changes of behaviour [31, 34, 56, 57]. These may be consistent changes in frequency or intensity of individual behaviours that remain relatively consistent with others [28, 45, 46] or individual changes of correlation among traits [31, 36, 44, 57].

The general aim of this study was to assess whether personality remains stable during the whole development. We hypothesise that the evolution of personality may be revealed by examining how proximate and ultimate factors influence the establishment of personality and its stability or changes during development. We address this question by a detailed description of both the differential and structural consistency. We have two basic assumptions about how personality is being shaped: (1) fixation of certain traits combinations is caused by proximate factors early in development (e.g., stress in the early ontogenetic life-stage [58–61], the initial body weight and maternal investment ([62, 63], but see [64]), and incubation temperature in reptiles ([9, 65, 66], but see [67]) and the traits are already associated in the juvenile stage and this is maintained during development, even though it might be quantitatively different [56]; (2) a structure of fixed traits changes over ontogeny—either through gradual changes in response to environmental challenges (foraging or predation risk [33]) or as a result of different selection pressures on adults and juveniles, favouring a different set of traits in different life stages [68–70].

As juveniles are confronted with different situations than adults, understanding personality development is crucial for better comprehension of ultimate and proximate mechanisms affecting personality [3]. The struggle to survive and reproduce are the main ultimate forces shaping animal personality traits. In their theoretical model, Wolf and his colleagues [71] demonstrated that evolution of animal personality might be explained by a trade-off between the current and future reproduction prospect, which leads to a behavioural diversification in populations, e.g., in risk-taking behaviour. This model predicts that shy and non-aggressive individuals should show slower development, delay reproduction, and spread the reproductive effort for latter stages of ontogeny and die later. On the other hand, bold and aggressive individuals are characterized by rapid maturation, early age of first reproduction, but earlier death [72]. Whenever animals face a similar type of trade-off, consequent development of personality types is expected over the ontogeny. Some species of boas solve a similar trade-off, they change reproduction according to the individual life expectation [73, 74]; changes in personality are also possible. Long-living species, such as boid snakes, have more opportunities for optimization of their behaviour. This optimization would be more easily observed in K-strategists, who also provide a longer window of opportunity for thorough testing of personality in consecutive life-stages from their birth/hatching to the adulthood. A high degree of precociality makes boas an ideal model group for studies of the ontogenetic personality pattern [73, 75, 76]. In our long-term study the inter-test intervals were standardized to life history of the studied species, the Northern common boa (*Boa imperator*), to cover the time from birth to sexual maturity of all individuals (more than four years).

Generally, we aimed to assess whether personality remains consistent in snakes during all important life stages, and to characterise any potential changes in personality we find (see above for predictions and consequences of theoretical models). If there is even a small change in personality during ontogeny, we expect to detect a change in the individual differential consistency. As some other longitudinal studies have already shown, personality becomes more stable during the animal’s lifetime [41, 48, 77]. We suppose that repeatability of the traits measured in earlier life stages would be lower. The concordance should be subject to a similar trend, but since it is calculated as a rank order coefficient, which is not as sensitive to changes in variability as repeatability, the difference between concordances in different life stages might not be as large as the difference between repeatability coefficients.

Specific aims of this study were to test the effect of age on personality traits and to explore potential differential and structural consistency. Concretely, we aimed to (1) verify the suitability of common personality tests for snakes and repeatability/concordance of the measured behavioural traits, (2) check the existence of context generality (behavioural syndrome) to find a correlation between principally different personality tests including a physiological response (heart and breath rate) to a stress situation. Finally, (3) we assessed the effect of hatchling body weight as a proxy of maternal investment on the personality structure as well as the effect of adult body weight as a causal factor influencing the adult personality.

## Materials and methods

### Experimental animals and their maintenance

Thirty captive-bred individuals of the Northern common boa, *Boa imperator*, Daudin 1803 (for systematic revision see [78]), were used in this study. The juveniles were obtained from two females, which shared the cage with three males. All individuals were of the second captive-bred generation, descendants of the founders with known region of origin (Chiapas, Mexico; for genetic analysis, see [79]). This arrangement made it impossible to discriminate exactly between the synchronized litters (both females gave births in two days), thus identify genetically similar individuals from the same litter. We sexed the snakes using a cloaca probing during the 5th and 13th month of age. Fourteen females and sixteen males were included in this study.

The Norther common boa is a live-bearing ectotherm species with embryos developing inside the mother’s body [80]. Pregnant females (mothers of experimental subjects) were housed in a breeding room with temperature varied from 26.5 to 31°C during January to June. Hotter basking sites (up to 44°C) were available because females tend to increase and stabilize body temperature in available temperature gradient during pregnancy [81]. However, embryos inside the snake body are developing in slightly different temperature gradient, which in another oviparous reptile species influences personality [9, 65, 66]. Young snakes were housed in a different room with temperature ranging between 28 to 30°C.

The snakes were housed individually in plastic boxes (first month, 50x30x25 cm) or glass cages (70x50x50; 130x120x100 cm), corresponding to the animal size under the standard conditions. They were regularly weighed (every week during the first year of life, less frequently later) and fed by laboratory mice or chicken with respect to their size (for details of both, see below). The water was provided ad libitum (for details, see [73]).

All the feeding experiments were conducted right before, or alternatively to regular feeding events. The relative total mass of the offered prey mostly represented 10–20% of the weight of the particular snake (5–25%). The feeding regime corresponded with the life stage and depended on the snake’s size and age; see below. No tests (except routine feeding trials) took place during the same day.

This study was approved by the Institutional Animal Care and Use Committee of the Charles University, Faculty of Science (Permit Number: 24773/2011-10001). The experiments were performed in accordance with the Czech law implementing all corresponding European Union regulations. All handling with snakes was carried out considerately to minimize suffering.

### Definition of life stages and feeding regime during the study

**Fig 1:**
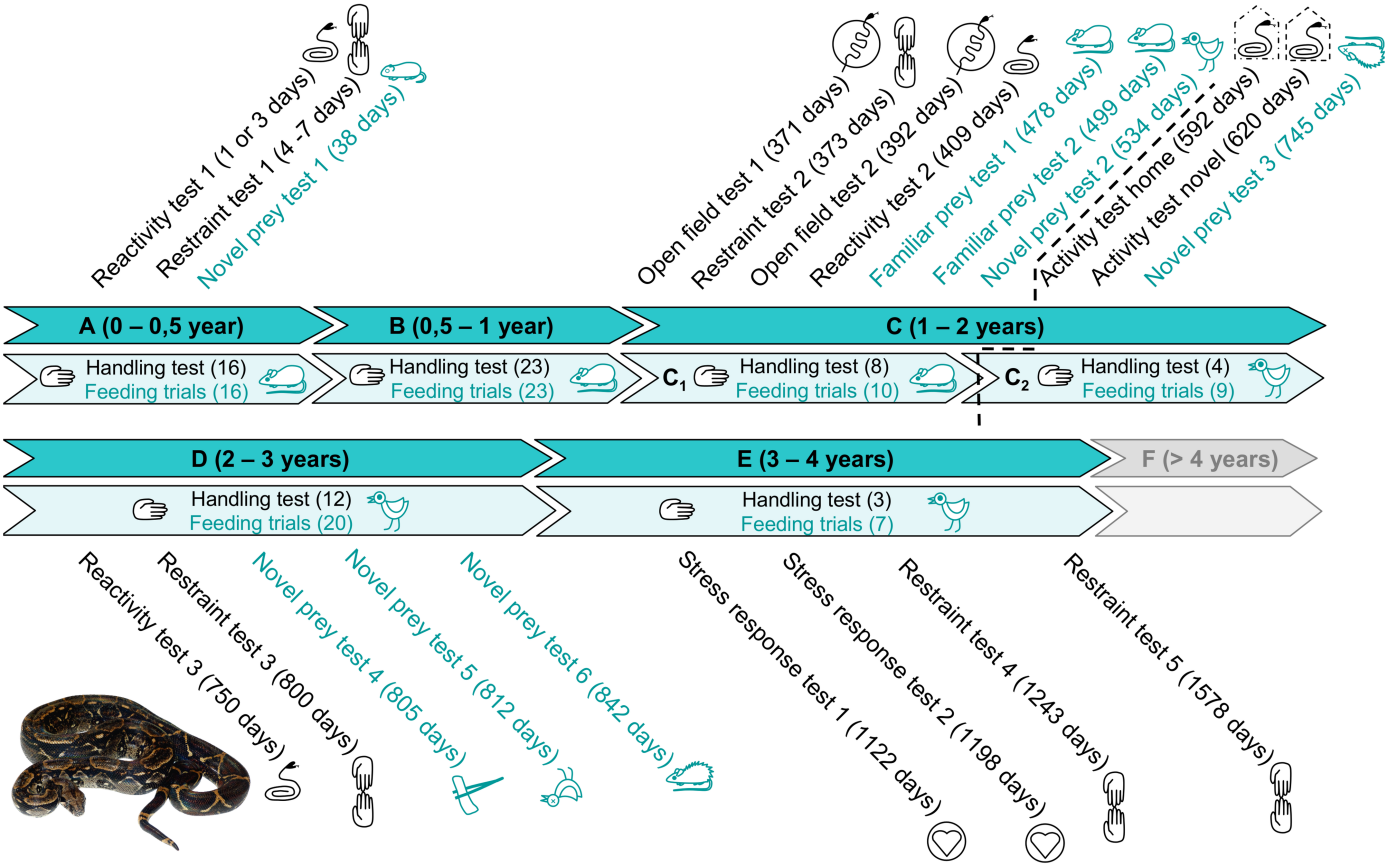
Timeline summarizing the whole experiment. A time table of particular tests administration at defined lifestages/feeding blocks. The life stages were adjusted to life history of the studied species (the Northern common boa, *Boa imperator*) to cover the time from birth to reaching sexual maturity of all individuals (more than four years).

To detect potential changes in behavioural traits accompanying rapid growth during the first two years [82], we set regular tests for assessment of feeding or defensive behaviours within half year periods during the first two years. As the body growth slows down at the age of two years, we adopted one-year periods for these tests up to the age of four. Furthermore, various additional personality tests were conducted throughout ontogeny in selected life stages to better describe personality axes, i.e., boldness, exploration, and activity [4, 24]. According to the feeding regime, prey type and growth rate, we divided four years of the study into five life stages and six feeding blocks (A—E), bordered as follows: 0.5, 1, 1.5, 2, 3 and 4 years of age (for the timeline, see Fig 1). As the time frame for snakes can be substantially different from those used by human experimenters, the life stages can be defined also in terms attributed to the snake life cycle.

The first block (life stage A, first six months of life) can be characterized as neonates. It includes weeks shortly after the birth, when the neonates gradually started to feed on mouse pups. When the snakes started to feed regularly, we routinely offered them small mice, but several reluctant feeders sometimes continued to accept only mouse or rat pups. All the thirty snakes accepted mice after three months of age. The feeding regime was one mouse per one, two, or three weeks.

The life stage B (0.5–1 year) corresponds with the younger juvenile phase. The animals were fed on mice and consumed practically all the offered prey, which corresponded with accelerated growth rate.

In older juveniles (life stage C, 1–2 years), the growth started to slow down. In addition, a transition from mice to chicken as a regular prey took place at 1.5 year and the effect of prey transition on feeding behaviour was checked. Thus, we split this life stage into two feeding blocks (C_1_ from 1 up to 1.5, C_2_ from 1.5 up to 2 years).

The next life stage, D (2–3 years), represented mainly subadult animals, but some individuals attained sexual maturity at the end of this period (3 years). At the end of life stage E (3–4 years), most of the individuals became sexually mature. In life stage F (4 years and more), all the animals were sexually mature, but still growing.

### Contexts of behaviour

#### Foraging and feeding context

The foraging and feeding behaviour (type and size of prey that is typically accepted) is one of the most prominent behavioural features in the life of a snake that may change during ontogeny [83]. On the other hand, some species show heritability of the feeding behaviour [84]. *Boa imperator* is an ambush predator, using a sit-and-wait strategy to capture the prey [80, 85]. Webb and his colleagues [86] discovered in other snake species that ambush foragers display slow life history (slower growth, later maturation) and their juveniles survive better. Also, to test the existence of the behavioural syndrome in Yucatan banded geckos (*Coleonyx elegans*), the authors used the feeding situation as a proxy of non-stressful situation, which motivates positively the activity of animals in familiar environment [65]. Thus, for all the above-mentioned reasons, we examined different variables representing important traits related to the foraging and feeding behaviour. Not only are these variables inherently connected to growth and ontogeny, but also present an important candidate for the assessment of personality traits.

Feeding trials. Snakes were regularly fed with laboratory mice or chicken (average mass 19.361 vs. 42.032 g, respectively) with respect to their size in regular intervals. All snakes were fed simultaneously at one day (see Fig 1). In two trials, we measured the catch latency of familiar prey. We also calculated the proportion of successful feeding trials (Prey acceptance) and an average score of catch latency (Index of catch latency). For more detailed definitions of the variables, see S1 File.

#### Boldness in novel prey context

Snakes are specialised predators hunting for big-bodied, but rarely encountered prey. Prey items regularly consumed by these cold-blooded predators are extremely large compared to their own body size (e.g., *Thamnophis sirtalis* accepts food representing 50% of their body size [87]). Considering that snakes are energy-savers minimizing their metabolic expenditures, such prey items are extremely valuable from the perspective of the utilised energy and metabolism. On the other hand, each foraging event is inevitably associated with considerable risks of injury or even death. It is because the snakes readily prey upon animals whose size approaches the upper limits of safe killing, swallowing and/or digesting [88, 89]. The decisions to hunt and/or consume a prey is thus of crucial importance for these animals and should be under a strong pressure of natural selection. Thus, bold-shy personality axis associated with foraging decisions can be reasonably expected in snakes.

Novel prey test. A novel prey is defined as a prey that was offered to a snake for the first time. The first prey after birth (a mouse pup) was also classified as a novel prey. Routine feeding with a familiar prey was replaced several times by offering an unfamiliar prey. Prior each experiment, the snakes were left undisturbed for about twenty minutes. (For details, see Fig 1, Table 1 and S1 File). A non-living prey (dead chicken, chicken neck, dead spiny mouse) was offered using long tweezers. If the snake did not show predatory behaviour during the first minute of food offering, the prey was left on the floor of the cage. We filmed these trials for 24 hours. We analysed the data about catch latencies of novel prey separately with respect to the development (the first experiments with juveniles were excluded) and/or according to the type of novel prey (live and dead), because it can have a great impact on feeding behaviour of the snakes [90].

**Table 1:** Summary of the behavioural tests used in this study of *Boa constrictor*.

Contexts	Behavioural tests	Repetition/ Blocks	Dependent variables	Variable type	Transformation
Foraging/ feeding	Feeding trials	9–23 trials/block	Prey acceptance	Percentage	Arcsin
		6 block repetitions	*Proportion of successful trials per block*		
		9–23 trials/block	Index of catch latency	Ordinal categories reflecting prey catching speed	Log
		6 block repetitions	*Average of speed categories per block*	*(1) until 3 s*, *(2) until 15 s*, *(3) until 1 hour*, *(4) within a day*	
		2 trials	Catch latency familiar	Latency (s)	Ln
			*Catching latency of familiar prey*		
Boldness	Novel prey test	5 trials	Catch latency novel	Latency (s)	Ln
			*Catching latency of novel prey*		
		3 trials	Capture success novel	Presence/absence	-
			*Catching latency of novel prey within 20 minutes*		
			Catch latency of novel live prey	Latency (s)	Ln
			*Catching latency of novel live prey (pup*, *mouse*, *chicken*, *spiny mouse)*		
			Catch latency of novel dead prey	Latency (s)	Ln
			*Catching latency of novel dead prey (dead chicken*, *dead spiny mouse*, *chicken neck)*		
Exploration	Open field test	4 trials	Movement latency	Latency (s)	Ln
			*Latency to start movement in open field (forced exploration)*		
			Time to leave the area of arena	Duration (s)	Ln
	Activity test A	1 trial	Percentage of movement familiar	Percentage	Arcsin
			*Proportion of movement in familiar environment for 24 hours*		
	Activity test B	1 trial	Percentage of movement	Percentage	Arcsin
			*Proportion of movement in novel environment for 20 hours*		
Agonistic	Handling test	3–23 trials/ block	Index of defensive behaviour during handling	Presence/absence	Ln
		6 block repetitions	*Hiss*, *attack*, *bite*		
	Reactivity test	3 trials	Number of tongue flicking	Quantity	Square root
			*As a response to nudging stimulus*		
			Movement	Presence/absence	-
			*Movement as a response to nudging stimulus*		
	Restraint test	5 trials	Occurrence of defensive behaviour	Presence/absence	-
			*As a response to forced immobility (hiss*, *attack*, *bite)*		
	Stress response test	2 trials	Heart rate resting and stress I	Number of heart beats	Ln
			*Before (resting) and after (stress) holding in hand head down as a stressful stimulus*		
			Breath rate resting and stress II	Number of breaths	Ln
			*Before (resting) and after (stress) holding in hand head down as a stressful stimulus*		
			Heart rate stress response	Number of heart beats	-
			*Difference between maximal (stress) and minimal (resting) heart beats*		
			Breath rate stress response	Number of breaths	-
			*Difference between maximal (stress) and minimal (resting) breath rate*		

The table presents a number of repetitions per block, explanation of the dependent variables, type of the variables and their transformation

We measured the latency to catch novel prey (Catch latency novel prey) and we also created a binary variable Capture success novel, which reflected whether or not the snake caught the prey in 20 minutes after introducing the novel prey. For more detailed definitions of the variables, see S1 File.

#### Context of exploration

Testing exploratory behaviour in novel environment (usually using by some variant of the open field) face several problems, because it is inherently connected with the activity of animals. However, in this situation many researchers claim that the tests measure emotional aspect of this behaviour (anxiety), especially in rodents [91], rather than exploratory tendencies. The open field was originally designed for rodents as a test of emotionality. It is based on placing the subject in a novel environment and measuring the elements of behaviour [92, 93]. Because of its relatively simple methodology, it has been used as a standard protocol measuring the exploratory behaviour in different animals. It has been used for lizards (*Zootoca vivivipara* [74]) and also for snakes (*Thamnophis melanogaster*, *T*. *sirtalis*, *T*. *butleri* [76] and *T*. *radix* [94]). Mayer and his colleagues [62] use the initiation of activity (emergence from a shelter in a novel arena) as a boldness measurement in hatchlings of the snake *Tropidonophis mairii*. The results from Chiszar and his colleagues [94] revealed that in the open field we can measure both, but we should differentiate between the free (voluntary emergence from the shelter) and forced (putting the animal into the arena without a shelter by hand) variant of the open field test.

Open field test. We adopted the general method of testing from Herzog and Burghardt [76], who successfully tested several species of the genus *Thamnophis* in an open field test (forced exploration assay). The arena consisted of a circle (with the diameter of 112 centimetres), marked on a floor of an unfamiliar room. The space was temperature-controlled (28°C). The experiment started by a gentle placement of a snake into the centre of the arena and ended when the animal's head crossed the border of the circle. The arena was washed (water and detergent) and deodorized (ethanol) before the next trial. The Open field test run in two sessions (marked here as 1 and 2)—the first one conducted at the age of 371 days, the second 24 days later; both sessions consisted of two trials following immediately after another. Thus, each individual participated in four trials (see Fig 1, Table 1, S1 File). We measured the latency to start moving after placing the snake in the arena and the latency to leave the arena.

Activity test in home and novel cage. The activity test was performed in a home and novel cage. The overall activity was evaluated from a video record. We used infrared sensitive camera and infrared reflector for recording during the dark phase of the day as well as during the day (we didn’t change the camera). Activity level in the home cage was recorded continuously for 24 hours. Recording of the activity inside the novel cage started by putting the snake gently into the cage. The recording had been running a shorter time (20 hours; from 3 p. m. to 10 a. m.), because the snakes were largely inactive during the light phase of the day. We have measured the percentage of active movement during the recording (variable Percentage of movement, for the detailed methodology see S1 File).

#### Agonistic behaviour context

The individual levels of agonistic behaviour can be assessed as: 1) the aggressiveness in within-species interactions (in geckos *Teratoscincus scincus*, *T*. *keyserlingii* [95], skink *Liopholis whitii* [31]) or using the shelter with the odour of conspecific (lizard *Zootoca vivipara* [8, 74, 96], here it is called also sociability); 2) the reaction to a simulated predator attack (in lizard *Lacerta monticola*, [97], in snakes various species of the genus *Thamnophis* [76, 84, 87, 98–100]). For the Northern common boa, the aggressive behaviour during interspecific interactions has not been reported in the literature so far, and we have never observed it in captivity. The only possible way for testing agonistic behaviour is to set up the experiments in antipredatory context. For that purpose, we designed three tests simulating a predator attack with increasing intensity: Reactivity test < Handling test < Restraint test. The test with medium intensity of predatory attack, the Handling test, was regularly used during the whole study to capture the potential development of agonistic behaviour (see Fig 1, Table 1, S1 File).

Reactivity test. A test simulating a non-specific predator attack [70, 84] was modified to be applicable to the boid snakes. The snakes were placed into a testing box sized either 15.5×15.5×11 cm (length, width, and height) or 30×17×14 cm (accordingly to the body size of the animal) and then they were left alone to adapt before the procedure. A black rounded container 54 × 32 cm (diameter and height) served as the testing arena. After a three hours’ acclimation period, the testing box was placed into the arena and the lid was slowly removed. The trial started by using the first stimulus—touch by a stick with rounded tip from a random direction in the first third of the body. We avoided touching the head (severe threat). The interval between the stimuli was 1 sec., each snake obtained ten stimuli, so the whole procedure lasted about 10 s. No handling occurred during this test.

All the trials were videotaped. We recorded the occurrence of any locomotor activity (Movement) and counted the number of tongue flicks (Tongue flicking). The defensive behaviour was so rare that we excluded it from the analysis. The Reactivity test was repeated three times: first several days after the birth, and then in one and two years of age (see Fig 1, Table 1, S1 File).

Handling test. Any manipulation with an animal by hand can be considered as a handling test. We had been routinely handling the snakes for the purposes of weighing and/or moving to another cage (for a number of handling tests in particular life stages see Fig 1). Weighing consisted of picking up the snake by hand, putting it into a box used for weighing and after that, taking the snake in hands again and returning it into the home cage. Such manipulation lasted for the shortest possible time, the snake was handled very gently and calmly and was not restrained or provoked to any action. The handling procedure was usually carried out by the same person (OS). The long-term monitoring of defensive behaviour refers to an occurrence of any type of the defensive behaviour during any handling trial in a particular block and is calculated as an *Index of defensive behaviour* per a block, similarly as the *Prey acceptance* (Table 1, Fig 1, S1 File).

Restraint test. Any manipulation, during which a snake is forced to stay motionless in a determined position can be considered a restraint test [101]. This fits to a situation during photographing and measuring in standard positions. These procedures required a transfer of the snake to an unfamiliar space and assistance of people, with whom the snake was not familiar. We focused on the defensive behaviour during this test. The Restraint tests were performed after the birth (0–2 days of age) and in one (373 days), two (800 days), three (1243 days) and four years of age (1578 days), see the Fig 1.

Stress response test. Because boid snakes are mostly passive animals, the observed inactivity or immobility may be misinterpreted as a resting state. Physiological traits are often used as correlates of personality, which can have substantial importance in the case of such inactive animals. Stress response test was designed for the detection of the reaction to stress using physiological values (cardio-respiratory parameters) not necessarily linked to an easily observable locomotion. We generally followed the rule for this test with respect to the physiology of ectotherms [102] and adapted them for measuring stress response in the Northern common boa as follows.

The animals were held on the knees of one of the experimenters during the measurements and gently restricted by hands. Both breathing and heartbeats were recorded simultaneously for three minutes and then, after one minute of more stressing activity (the animal was held in hand head down, provoked to move), it was measured once again for more three minutes. The first measurement started immediately after removing the animal out of a cage (the rates presumably closest to the resting values); only in several extremely reactive animals, we had to wait until they calmed down. We repeated the same procedure after three months (11 weeks) during the same time of the day and under the same temperature conditions (from 25.6 to 26.7°C). We obtained twelve minutes of usable records for each individual at total, six minutes before the forced activity (initial values) and six minutes after (stress values). For a summary of variables derived from the Stress response test, see Table 1 and S1 File.

### Methods of statistical testing

#### Differential consistency

Repeatability. We calculated repeatability as a measure of the differential consistency and to assess whether the individual’s variance of behaviour was significantly smaller than the overall variance of our sample. In other words, whether the animals are consistently different from each other. Kendall’s coefficient of concordance (W) can also be conducted to assess the agreement across the repeats of the test, but repeatability provides us with stricter criteria than the Kendall’s W, which only uses the rank order, while repeatability compares the intra- and inter-individual variability. Repeatability was calculated according to the methods described in Nakagawa and Schielzeth [39]. We used untransformed data in most of the variables for the calculation to ensure unchanged variation [103] during the analyses, which we conducted using the rptR package in R [104]. We used a GLMM model (command rpt.poisGLMM.multi) to analyse the repeatability of for count data (e.g., the latency, heart and breath rate data) and GLMM model for binary data (command rpt.binomGLMM.multi). When the data were neither count nor binary, we used their transformed forms in a LMM model (method REML, command rpt.remlLMM). Table 2 gives a more detailed overview of the methods and S2 Table contains the source data.

**Table 2:** Review of results and statistical methods employed in the present study of the Northern common boa.

Behavioural tests	Dependent variables	Context	Between-trial interval	Function	Statistical package and approach	Repeatability (R)	Confidence intervals 95% (CI)	Statistical significance (P)
Feeding trials	Prey acceptance	F	weeks (mean 18 days)	remlLMM	R, REML method	0.113	0–0.25	0.014
	Index of catch latency	F	weeks (mean 18 days)	remlLMM	R, REML method	0.408	0.23–0.56	<0.001
	Catch latency familiar	F	weeks (21 days)	remlLMM	R, REML method	0.626	0.33–0.80	<0.001
Novel prey test	Catch latency novel (all prey together^a^)	B	weeks—months (mean 161 days)	pois.GLMM.multi	R, PQL method, loglink	NS	NS	NS
	Capture success novel	B	months (mean 402 days)	binom.GLMM	R, PQL method, logitlink	NS	NS	NS
	Catch latency novel (live prey^a^)	B	months (308 days)	pois.GLMM.multi	R, PQL method, loglink	NS	NS	NS
	Catch latency novel (dead prey)	B	weeks (mean 33 days)	pois.GLMM.multi	R, PQL method, loglink	NS	NS	NS
Open field test	Movement latency (all trials)	E	24 days	pois.GLMM.multi	R, PQL method, loglink	0.306	0.14–0.54	0.001
	Movement latency (1a and 1b)	E	10 minutes	pois.GLMM.multi	R, PQL method, loglink	0.517	0.31–0.77	0.007
	Movement latency (2a and 2b)	E	10 minutes	pois.GLMM.multi	R, PQL method, loglink	NS	NS	NS
	Time to leave the area of arena (all trials)	E	24 days	pois.GLMM.multi	R, PQL method, loglink	0.12	0–0.316	0.077
	Time to leave the area of arena (1a and 1b)	E	10 minutes	pois.GLMM.multi	R, PQL method, loglink	0.401	0.06–0.68	0.007
	Time to leave the area of arena (2a and 2b)	E	10 minutes	pois.GLMM.multi	R, PQL method, loglink	0.388	0.15–0.69	0.068
Activity test	Percentage of movement	E	one month	remlLMM	R, REML method	NS	NS	NS
Handling test	Index of defensive behaviour	A	weeks (mean 23 days)	binom.GLMM	R, PQL method, logitlink	0.385	0.12–0.57	0.001
Reactivity test	Tongue flicking	A	months (mean 374 days)	GLMM.multi	R, PQL method, loglink	0.165	0–0.39	0.046
	Movement	A	months (mean 374 days)	GLMM.add	MCMC method	NS	NS	NS
Restraint test	Occurrence of defensive behaviour	A	months (mean 394 days)	binomGLMM.multi	R, PQL method, logitlink	NS	NS	NS
Stress response test	Heart rate within the test I (resting)	A	3 minutes	GLMM.multi	R, PQL method, loglink	0.776	0.35–0.75	0.001
	Heart rate within the test II (resting)	A	3 minutes	GLMM.multi	R, PQL method, loglink	0.849	0.46–0.80	0.001
	Heart rate within the test I (stress)	A	3 minutes	GLMM.multi	R, PQL method, loglink	0.704	*0*.*17–0*.*63*	0.001
	Heart rate within the test II (stress)	A	3 minutes	GLMM.multi	R, PQL method, loglink	0.562	*0*.*0–0*.*38*	0.001
	Breath rate within the test I (resting)	A	3 minutes	GLMM.multi	R, PQL method, loglink	0.747	0.57–0.88	0.001
	Breath rate within the test II (resting)	A	3 minutes	GLMM.multi	R, PQL method, loglink	0.889	0.79–0.95	0.001
	Breath rate within the test I (stress)	A	3 minutes	GLMM.multi	R, PQL method, loglink	0.756	0.58–0.86	0.001
	Breath rate within the test II (stress)	A	3 minutes	GLMM.multi	R, PQL method, loglink	0.643	0.44–0.78	0.001
	Heart rate between the tests I vs II (resting)	A	10 minutes	GLMM.multi	R, PQL method, loglink	0.525	0.33–0.66	0.001
	Heart rate between the tests I vs II (stress)	A	10 minutes	GLMM.multi	R, PQL method, loglink	0.588	0.19–0.54	0.001
	Breath rate between the tests I vs II (resting)	A	10 minutes	GLMM.multi	R, PQL method, loglink	0.621	0.42–0.76	0.001
	Breath rate between the tests I vs II (stress)	A	10 minutes	GLMM.multi	R, PQL method, loglink	0.501	0.31–0.65	0.001
	Heart rate stress response	A	3 months	pois.GLMM.multi	R, PQL method, loglink	0.303	0–0.60	0.029
	Breath rate stress response	A	3 months	pois.GLMM.multi	R, PQL method, loglink	0.482	0.15–0.75	0.001

The type of models, procedures and software are provided for each analysis. Abbreviations of contexts: (F) Foraging/feeding context, (B) Boldness in novel prey context, (E) Context of exploration, (A) Agonistic behaviour context. N.S. indicates non-significant results.

^a^ The analyses were conducted without the first feeding (pup).

Consistency across life stages. Repeatability provided us with the comparison of intra- and inter-individual variability, however it does not provide direct information about the changes in the rank order of the animals. A rank order correlation coefficient can help us assess if the behaviour is stable across life stages or if the relative intensity of the behaviour (when compared to other subjects) significantly changes. For this purpose, we applied the Kendall’s coefficient of concordance (W), which is a non-parametric test enabling us to calculate the difference between rank orders extracted from multiple repeats (Statistica Help). Because there were reasons to expect the behaviour during the first year of life to be different (as mentioned in Introduction), we also applied the Kendall’s coefficient of concordance to the data excluding the first year.

Development of selected personality traits. We analysed the effect of the stage of life, sex and individuality with the mixed-effects models. For the data with normal distribution (Prey acceptance, which was square-root arcsin transformed; Index of catch latency, log transformed) we used the marginal model (gls function under nlme package in R, exchangeable correlation structure) with the life stage and sex as a fixed effect and the individual as a random effect.

For the binary data (Capture success novel, Movement, Occurrence of defensive behaviour and Index of defensive behaviour), we employed statistical models accounting for repeated testing of the same individual as implemented in the geeglm package under the R-environment (R-project) with the individuality of the snake as a random effect. For Tongue flicking in Reactivity test, we used the generalized marginal model for Poisson data (glm function in R) with the individual as a fixed effect.

Context generality as correlation structure among set of tests at one life stage. To identify the multivariate axes in life stages A, C_1_, D, and E, we used the Principal Component Analysis (PCA) in Statistica, version 6 [105] that also allowed us to extract more exact individual scores for each principal component (for source data see S2 Table). We applied PCA analysis on the set of tests (the Feeding trials, Handling test, Restraint test, Reactivity test and Novel prey tests) conducted at one life stage to calculate context generality. A reduced set of tests was also conducted for adults (the life stage E), where the Reactivity and Novel prey tests were not included. As the same (similar in the case of E) set of tests was replicated four times (life stages A, C_1_, D, E), we calculated four independent PCA analyses. Two or three main factors were extracted. The number of principal components retained for the analysis was based on eigenvalues >1, a scree plot, the amount of variation explained by the factor and the interpretability [31, 106]. The first aim was to compare whether the extracted multivariate axes were related to a specific set of variables measured in different tests (test of contextual generality). As the PCA is mainly explorative statistical method, we confirmed the relationship among variables measured in different tests (different context, e.g., the Feeding trials and the Open field) that were highly correlated among particular multivariate axes. The Spearman’s correlation tests were used because some of our raw behavioural data were slightly skewed (for PCA were normalized, see Table 1).

Further, we examined whether the combination of variables contributing to the particular multivariate axes (PC1, PC2 and PC3) (behavioural profile) remained the same or not in these particular axes calculated for each life stage (PCA for stages A, C_1_, D, and (E)). We explored whether the correlations between the behavioural profile and the multivariate axes were qualitatively constant across the life stages (structural consistency), or if there was an apparent change in the correlational matrix related to the developmental process (also, see below).

#### Context generality and structural consistency

Our first approach to reveal the structural consistency was checking the mutual correspondence of the scores derived from multivariate axis from PCAs of the same set of behaviours in selected life stages (A, C_1_, D, (E see above)). Moreover, we checked whether there was an apparent change in the correlational matrix related to the developmental process. We used Pearson’s correlation coefficient to assess the correlation between the PCA scores for the individuals in particular life stages in A, B, C, D, E. We adopted this approach to illustrate the potential for structural consistency of personality in snakes, which needs proper testing in the future. The limitation of the Pearson’s r is that the levels of significance of such tests are questionable because multiple testing can lead to artificially “significant” results (see also [9]), but the relationship among multivariate axes across tests and across life stages is more clear. Moreover, Pearson’s r and p—values provide important basic information about the patterns of covariation among multivariate axes derived in separate life stages.

The second approach was to determine the mutual correspondence of all the conducted tests across all life stages. We employed the factor analysis in Statistica software with principal axis method of factor extraction to assess the main axes behind the overall behavioural variability. Furthermore, we analysed the same data using cluster analysis (Ward’s method using Pearson’s r) to assess the relationship between variables associated with one axis across all life stages.

#### Body weight as a proximate factor involving personality

We used individual factor scores for Factor 1, Factor 2 and Factor 3 to test the influence of body size on the correlational structure of behaviours across all life stages (source data in S3 Table). The Pearson’s r was used to assess the correlation between the individual factor scores and snakes’ body weight at birth (initial) and at four years (final).

## Results

### Differential consistency

#### Foraging and feeding context

**Fig 2:**
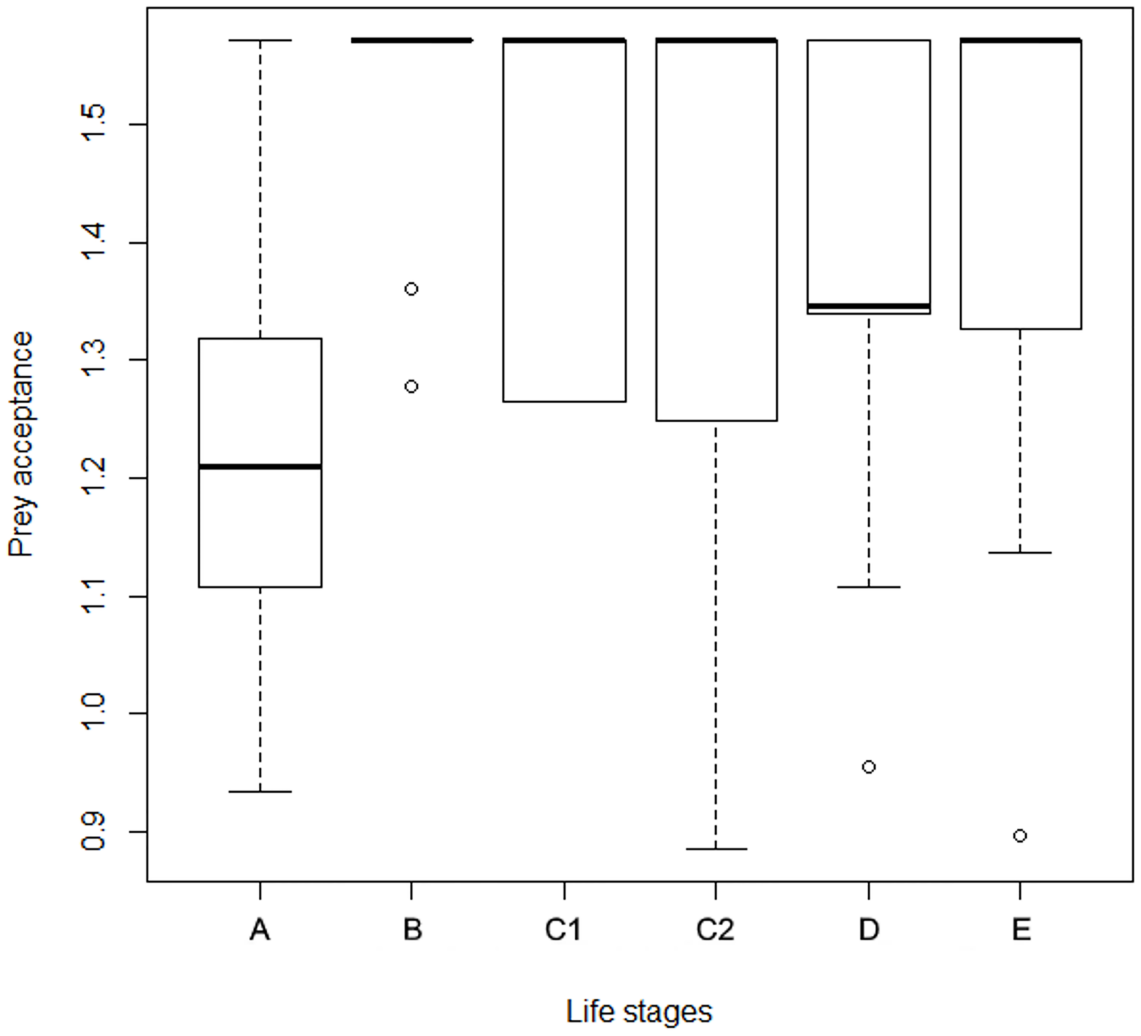
Development of Prey acceptance. Development of variable Prey acceptance measured in the Feeding trials, calculated as an index from regular feeding trials conducted during the whole experiment in six life stages A (0–0.5 years), B (0.5-1years), C_1_ (1–1.5 years), C_2_ (1.5–2 years), D (2–3 years) and E (3–4 years). The Prey acceptance in early development is significantly different from the subsequent life stages, yet this behaviour is significantly repeatable (R = 0.113, p = 0.014) and fairly consistent as revealed by Kendal’s test (Kendal’s W = 0.367).

In this context, all variables (Prey acceptance, Catch latency of familiar prey, Index of catch latency) showed significant repeatability. Prey acceptance and Index of catch latency both had significant concordance across life stages (W = 0.367 and W = 0.386, respectively), however after excluding the first year, the concordance of Prey acceptance was no longer significant. For the estimates of repeatability, see Table 2, for the Kendall’s coefficient of concordance, see S1 Table. Both Prey acceptance and Index of catch latency were measured in multiple life stages, therefore we also analysed the development of these traits with a marginal model (gls function in R, life stage and sex as a fixed effect, identity of the individual as a random effect). Prey acceptance was square root arcsin transformed. The life stage had a significant effect (F = 18, p = 0.0001), but the sex did not (p = 0.417). All the life stages (B-E) were significantly different from the first life stage (Fig 2). Index of catch latency was log-transformed. The effect of life stages was significant (F = 17; p < 0.001), the sex was not (p = 0.644). The stages C_1_ and D significantly differed from the first stage (A).

#### Boldness in novel prey context

Boldness was represented by novel prey trials and mostly by measures of catch latency of novel prey. None of the variables had significant repeatability, however they all had good concordance (ranging from W = 0.229 to W = 0.566). For the estimates of repeatability, see Table 2, for the Kendall’s coefficient of concordance, see S1 Table. We have tested the effect of three life stages A, C and D and sex on Capture success of novel prey. We employed statistical models accounting for repeated testing of the same individual as implemented in the geeglm package under the R-environment with the snake’s individuality as a random effect. The effect of life stage was significant (Residual deviance = 15771, p < 0.0001), the effect of sex was not. Life stage D was significantly different from life stage A.

#### Context of exploration

The variable Time to leave the arena from open field test mostly had significant both repeatability and concordance (r = 0.306; W = 0.232). The variable Movement latency had significant repeatability and concordance overall, however when comparing only the two trials performed in the same day, the concordance was not significant and repeatability was significant only in the first session (1a and 1b, r = 0.517). The activity test didn’t show significant repeatability, but the concordance was significant. These tests were not conducted in different life stages; therefore, we could not test the effect of development on these traits. For the estimates of repeatability, see Table 2, for the Kendall’s coefficient of concordance, see S1 Table.

#### Agonistic behaviour context

Most of the variables measured in this context were repeatable, but the concordance was not significant in most cases. The best repeatability and concordance were found in variables from the Stress response test, while the Restraint test show neither significant repeatability, nor significant concordance. For the estimates of repeatability, see Table 2, for the Kendall’s coefficient of concordance, see S1 Table. Handling, Reactivity and Restraint tests were all performed across different life stages and therefore we could analyse the effect of development. We employed a generalized marginal model (geeglm function in R for binomial data) with the life stage and sex as fixed factors and the individual as a random effect.

In the Reactivity test, neither the effect of life stage nor sex was significant. The model for the second variable in the Reactivity test (Tongue flicking) was calculated as a generalized marginal model (glm function in R for Poisson data) with the sex, life stage and individual as fixed effects. Individuality was the only significant factor (Residual deviance = 149, p < 0.0001).

In the Handling test, we measured the Index of defensive behaviour in six life stages (A- E). We adopted the same models we had already used for the occurrence of Movement in the Reactivity test. Neither the effect of sex nor life stage were significant.

In the Restraint test, we tested the Occurrence of defensive behaviour with the same models already used for the Movement in the Reactivity test. Neither the sex, nor life stage A, C, D, E and F were significant.

### Context generality

#### Context generality as a correlation structure among the set of tests at one life stage

**Fig 3:**
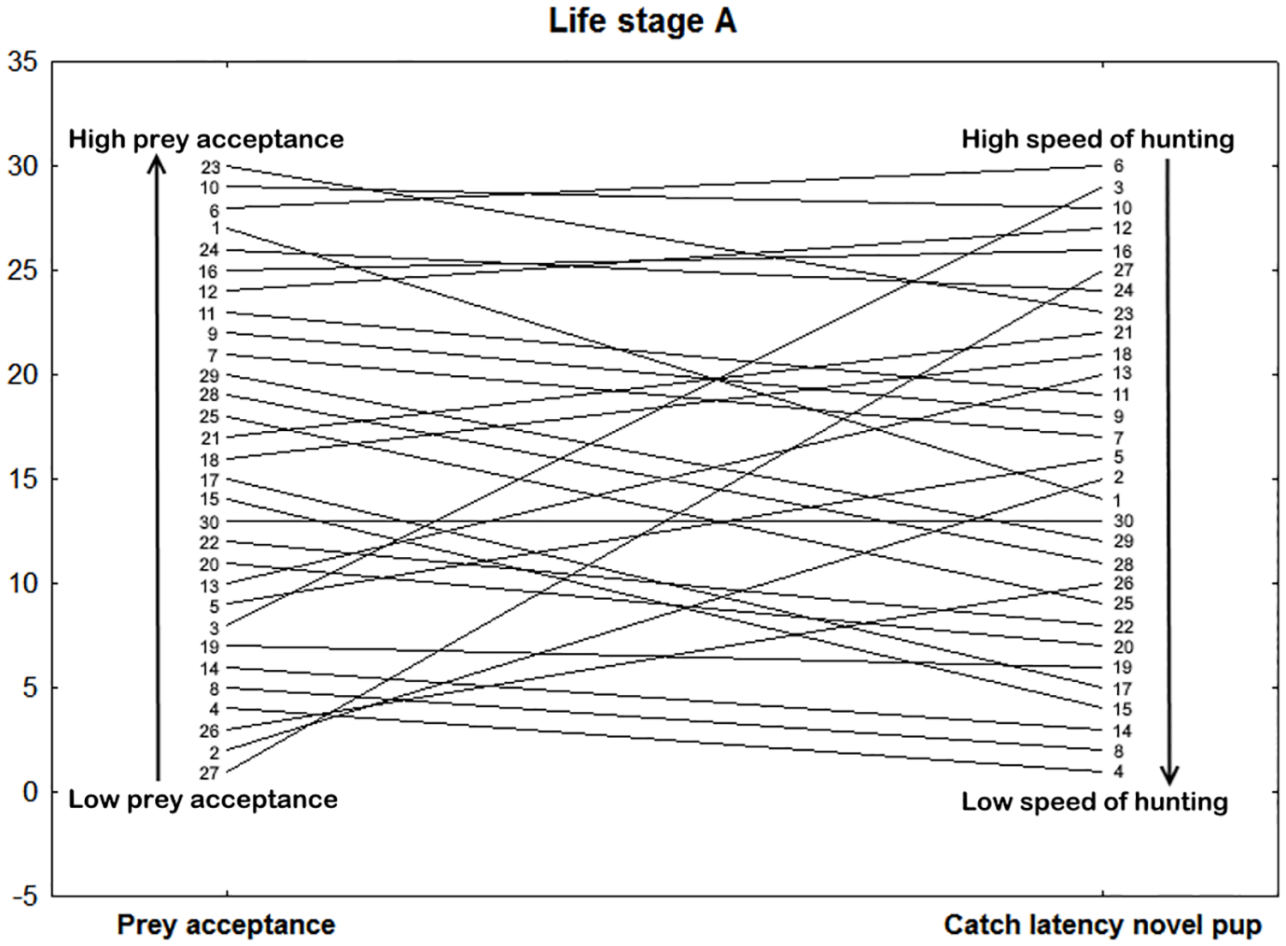
Example of context generality in juvenile life stage A (0–0.5 year). Visualization of the context generality between the Prey acceptance and Catch latency measured in the Feeding trials or Novel prey test, respectively (Spearman’s correlation coefficient = -0.498, p = 0.005). Vertical axis represents rank of the individual ordered according to a particular trait. Catch latency rank order was inverted, so that the lowest Catch latency (i.e. the fastest animals) are in the upper right part of the figure. The lines represent the rank stability/change for each snake in two different contexts. The numbers refer to identity of individual snakes (1–30). Therefore, in the ideal situation where all animals are perfectly consistent, all slopes would be zero (e.g. individual number 30). Snake number six has the third biggest proportion of successful feeding trials per block (Prey acceptance) with the shortest Latency to catch the novel prey (the fastest foraging decision).

In juveniles, the first axis (PC1A) was associated with variables from agonistic context (Index of defensive behaviour and Occurrence of defensive behaviour). These two variables (raw data) from different tests were also positively correlated (Spearman’s coefficient, r = 0.484, p = 0.007). The variable from feeding/foraging context (Prey acceptance) was associated with this axis only slightly (for loadings see Table 3). The second axis (PC2A) was also associated with agonistic context (Movement and Tongue flicking). The third axis (PC3A) was related to feeding behaviour (Prey acceptance and Catch latency novel). These two variables (raw data) from different tests correlated negatively (Spearman’s coefficient, r = -0.498, p = 0.005), thus, the juveniles that frequently accepted familiar prey also had short latencies in the Novel prey test (for a visualization of individual ranks ordered see Fig 3).

**Table 3:** Principal component analysis (PCA) of behavioural data across the life stages.

Loadings	Life stage A			Lige stage C_1_			Life stage D		Life stage E	
	PC1	PC2	PC3	PC1	PC2	PC3	PC1	PC2	PC1	PC2
Prey acceptance	0.375	-0.153	**-0.749**	**0.776**	-0.284	0.042	**0.601**	-0.329	-0.131	**-0.836**
Index of catch latency	0.209	0.335	0.416	-0.463	**0.672**	-0.012	**-0.719**	0.284	-0.336	**0.685**
Catch latency novel	-0.011	-0.142	**0.811**	**-0.873**	0.101	0.021	**-0.631**	0.074		
Index of defensive behaviour	**0.853**	0.218	0.069	**0.648**	0.492	0.071	-0.028	**-0.899**	**0.879**	0.135
Movement	-0.041	**-0.807**	0.166	0.014	**-0.834**	0.080	**0.872**	0.141		
Tongue flicking	0.011	**0.749**	0.193	0.174	0.085	**-0.823**	**0.582**	0.010		
Occurrence of defensive behaviour	**0.824**	-0.095	-0.193	0.215	0.012	**0.772**	0.159	**-0.816**	**0.767**	-0.400
Variance explained	1.593	1.426	1.499	2.076	1.488	1.288	2.402	1.688	1.490	1.346
Percentage explained	26.537	22.760	15.251	31.657	19.995	17.670	37.139	21.288	43.388	27.509
Eigenvalue	1.8576	1.5932	1.0675	2.216	1.3996	1.2369	2.5997	1.4902	1.7355	1.1003

The loadings of the variables included in PCA analyses conducted separately for each life stage: A (0–0.5 years), C_1_ (1–1.5 years), D (2–3 years) and E (3–4 years). Variable loadings larger than 0.5 are bold.

In older juveniles, the first axis (PC1C_1_) correlated with a mix of variables related either to agonistic behaviour or to the feeding/foraging context. First axis was also negatively correlated with behaviour reflecting boldness (Catch latency novel). The raw behaviours measured in the Feeding trials (Prey acceptance) correlated negatively with speed of hunting measured in the same tests (Index of catch latency, Spearman’s coefficient, r = -0.375, p = 0.0041), and the Novel prey test (Catch latency of novel prey (chicken), Spearman’s coefficient, r = -0.68, p < 0.0001). The second axis (PC2C_1_) was again consisted of a mixture of agonistic behaviours (Movement, Index of defensive behaviour) and one parameter of the feeding behaviour (Index of catch latency). In other words, animals that were highly moving in the Reactivity tests were those with low scores of agonistic behaviours in the Handling test and with longer hesitation to attack the prey in the Feeding trials. However, there was no significant Spearman correlation among these variables when calculated as raw data. The third axis also consisted of agonistic behaviours (for loadings see Table 3).

**Fig 4:**
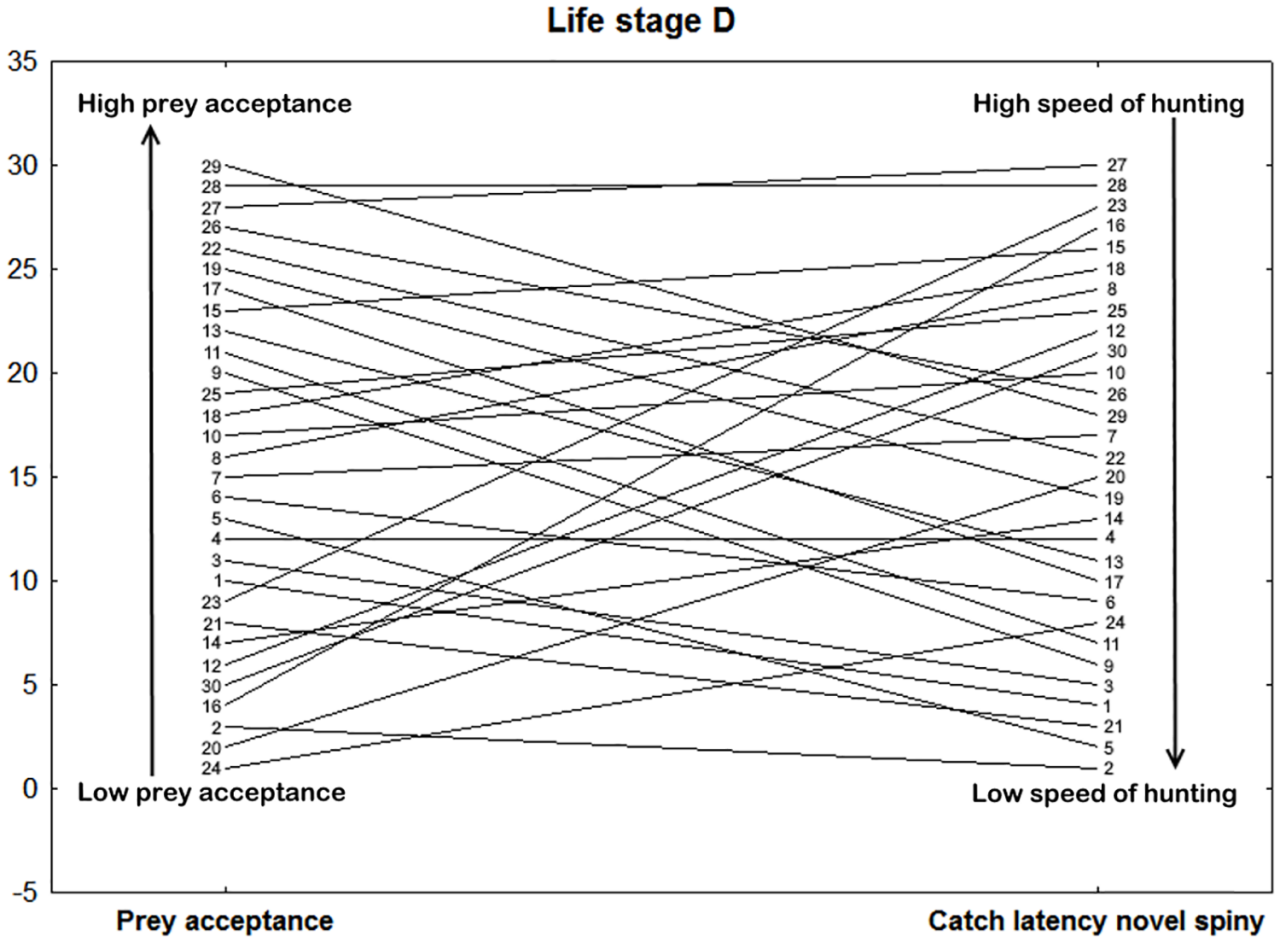
Example of context generality in subadults, life stage D (3–4 year). Visualization of the context generality between the Prey acceptance and Catch latency measured in the Feeding trials or Novel prey test, respectively (Spearman’s correlation coefficient = -0.094, p = 0.619). Vertical axis represents the rank of the individual ordered according to a particular trait. Catch latency rank order was inverted, so that the lowest Catch latency (i.e. the fastest animals) are in the upper right part of the figure. The lines represent the rank stability/change for each snake in two different contexts. The numbers refer to identity of individual snakes (1–30). Therefore, in the ideal situation where all animals are perfectly consistent, all slopes would be zero (e.g. individual number 4). Snake number twenty-seven has the third biggest proportion of successful feeding trials per block (Prey acceptance) with the shortest Latency to catch the novel prey (the fastest foraging decision).

**Fig 5:**
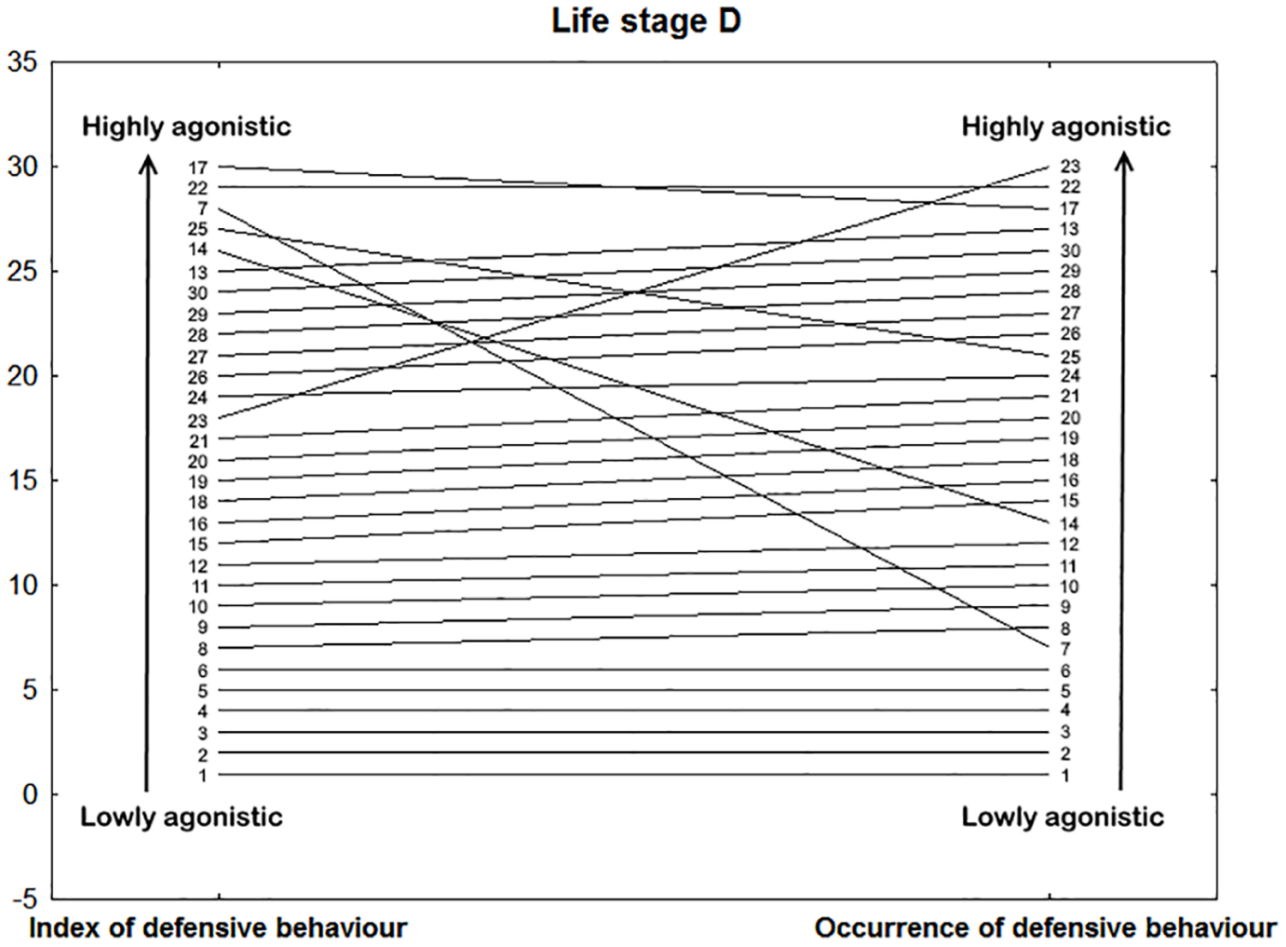
Example of context generality in subadults, life stage D (3–4 year). Visualization of the context generality between the Index of defensive behaviour and Occurrence of defensive behaviour measured in the Handling test or Restraint test, respectively (Spearman’s correlation coefficient = 0.539, p = 0.002). Vertical axis represents the rank of the individual ordered according to a particular trait. The lines represent the rank stability/change for each snake in two different contexts. The numbers refer to identity of individual snakes (1–30). Therefore, in the ideal situation where all animals are perfectly consistent, all slopes would be zero (e.g. individuals number 1–6). Snake number one has the smallest Index of defensive behaviour as well as the Occurrence of defensive behaviour.

In subadults, the first axis correlated with parameters measured in several contexts (Prey acceptance, Index of catch latency, Catch latency novel, Movement and Tongue flicking; for loadings see Table 3). For a correlation of individual ranks ordered according to the two traits (Prey acceptance and Catch latency) see Fig 4. The raw data about Prey acceptance from the Feeding trials are slightly correlated with those from the Reactivity test (Tongue flicking; only on p level 0.1, Spearman’s coefficient, r = -0.302, p < 0.1 and Movement, Spearman’s coefficient, r = 0.381, p = 0.038). Furthermore, two different behavioural variables (raw data) from the Feeding trials (Prey acceptance and Index of catch latency) were negatively correlated (Spearman’s coefficient = -0.488, p = 0.006). The second axis correlated with agonistic behaviours (Index of defensive behaviour and Occurrence of defensive behaviour). The latter two variables (raw data) from different tests were positively correlated (Spearman’s coefficient, r = 0.539, p = 0.002). For a visualization of individual ranks ordered according to these two traits see Fig 5.

A reduced set of tests was also conducted for adults, in which the Reactivity and Novel prey tests were not included. We calculated PCA analysis for this age as well. The extracted multivariate axes (for loadings see Table 3) correlated either with agonistic behaviours (PC1E, Index of defensive behaviour and Occurrence of defensive behaviour), or behaviours connected to the Feeding trials (PC2E, Prey acceptance and Index of catch latency). These two multivariate axes were not fully comparable with the previous analyses and were used for depicting the overall developmental effect only.

### Structural consistency

#### Stability or development of correlational structure over time

As mentioned above, the individual PC1, PC2, and PC3 (where applicable) scores computed for contextual generality in each of the analysed life stages (A, C_1_, D, E) were further analysed. We then calculated Pearson’s r reflecting the similarity among axes: adults PC1E was highly correlated with sub adult’s PC2D (Pearson’s r = -0.667, p < 0.0001). The second adult multivariate axis PC2E was correlated with both sub adult’s multivariate axes (PC1D Pearson’s r = -0.498, p = 0.005; PC2D Pearson’s r = 0.42, p = 0.02) and slightly with those for older juveniles (PC1C_1_ Pearson’s r = -0.427, p = 0.019; PC2C_1_ Pearson’s r = 0.385, p = 0.036). The sub adult’s PC1D (mainly reflecting foraging/feeding behavioural profile) was negatively correlated with the axes from the life stage C (older juveniles) with a mixed behavioural profile (PC2C_1_ Pearson’s r = -0.476, p = 0.008; PC3C_1_ Pearson’s r = -0,375, p = 0.041). Generally, the stages reflecting the change of a correlational structure in this analysis were those generating uncorrelated or slightly correlated PC scores (thus reflecting change), mainly juveniles and older juveniles (A and C_1_), see also Table 3.

#### Overall correlational structure of all tests and the additive role of particular life stages

We used factor analysis to determine the three main groups of inter-correlated variables. The primary Factor 1 of overall analysis (16.3% of the total variation) was the best correlated with Feeding trials, especially with quick hunting, which increased with age (Index of catch latency, for juveniles (A) r = 0.44 (minimum), for subadults (D), r = 0.88). Also, the Factor 1 moderately correlated with quick hunting in Novel prey tests in all life stages (Catch latency novel prey, for older juveniles (C_2_) r = 0.37; for subadults (D), r = 0.49) except of juveniles (A). However, the intensity of movement measured in the Reactivity test correlated negatively with this factor, but only in subadults (D). So, the highly moving (escaping) subadult animals were those who slowly attacked regular or novel prey (Movement, for subadults (D), r = -0.66). One behaviour from the Open field test also reflected a similar trend in older juveniles (Time to leave the arena, the first trial, for older juveniles (C_1_), r = -0.41). Behaviours associated with the first factor were inter-correlated across different tests and across different life stages and showed the foraging/feeding behavioural profile, probably fully established in subadults (D).

Factor 2 of overall analysis (10.3% of the total variation) was highly correlated with defensive behaviours from the Handling test, reflecting low intensity of aggressive behaviour in the juveniles (Index of defensive behaviour for juveniles (A), r = 0.44) and also in adults (E, r = 0.33), which is surprising when compared with the high scores in subadults (D, r = 0.7). The intensity of aggressive behaviour measured in the Restraint test is also high in subadults (Occurrence of defensive behaviours in subadults (D), r = 0.4) and adults (E, F, r = 0.58). Surprisingly, the highly aggressive juveniles also showed a higher frequency of tongue flicking measured in the Reactivity test (for juveniles (A), r = 0.43). Results of the Stress response test measured in adult snakes showed lower heart rates in the highly aggressive animals (Heart rate stress response, for adults (E), r = -0.59), but also higher breath rates (Breath rate stress response = 0.47). Behaviours associated with the second factor were highly inter-correlated across different tests and across different life stages, showing agonistic behavioural profile, which is probably present in juveniles and maintained to the adulthood.

Factor 3 of the overall analysis (8.4% of the total variation) was associated with behaviours reflecting an exploration activity measured in the Open field test (Movement latency, for older juveniles (C_1_), r = 0.0.58 (maximum), Time to leave the area of arena, r = 0.58 (maximum)) and Tongue flicking (r = -0.54 in subadults, D). These behaviours thus clearly represented the activity-exploratory behavioural profile, but in the current dataset we have had a limited number of tests that would measure it. For more detailed results, see Table 4.

**Table 4:** Factor analysis (FA) of the behavioural data.

Behavioural tests	Variables	Life stage	Factor 1	Factor 2	Factor 3
Feeding trials	Prey acceptance	A	0.135	-0.086	**0.483**
		B	0.105	0.065	0.256
		C_1_	**-0.506**	0.184	0.294
		C_2_	-0.292	0.218	**0.316**
		D	-0.383	0.387	-0.010
		E	-0.124	0.456	0.183
	Index of catch latency	A	**0.448**	0.144	-0.196
		B	**0.680**	0.073	-0.069
		C_1_	**0.836**	0.020	-0.128
		C_2_	**0.496**	0.120	0.150
		D	**0.884**	-0.119	0.092
		E	**0.760**	-0.196	-0.126
	Catch latency familiar 1	C_1_	**0.792**	-0.022	0.073
	Catch latency familiar 2	C_1_	**0.779**	-0.059	0.029
Novel prey test	Catch latency novel pup	A	*-0*.*290*	*0*.*144*	*-0*.*228*
	Catch latency novel chicken	C_1_	***0*.*454***	*-0*.*210*	*-0*.*182*
	Catch latency novel chicken neck	D	***0*.*491***	*-0*.*012*	***0*.*365***
	Catch latency novel dead chicken	D	***0*.*450***	*-0*.*351*	***0*.*408***
	Catch latency novel spiny mouse	D	***0*.*387***	*-0*.*215*	*0*.*078*
	Catch latency novel dead spiny mouse	C_2_	***0*.*370***	*0*.*136*	*-0*.*180*
Open field test	Movement latency 1a	C_1_	-0.177	0.090	**0.302**
	Movement latency 1b	C_1_	-0.245	0.192	**0.579**
	Movement latency 2a	C_1_	*-0*.*032*	*0*.*075*	***0*.*566***
	Movement latency 2b	C_1_	*0*.*025*	***0*.*360***	*0*.*034*
	Time to leave the area of arena 1a	C_1_	**-0.406**	-0.137	**0.579**
	Time to leave the area of arena 1b	C_1_	-0.246	0.338	**0.491**
	Time to leave the area of arena 2a	C_1_	*-0*.*096*	*-0*.*036*	***0*.*481***
	Time to leave the area of arena 2b	C_1_	*-0*.*055*	*0*.*098*	*-0*.*156*
Activity test home	Percentage of movement familiar	C_2_	***0*.*313***	*0*.*197*	*-0*.*149*
Activity test novel	Percentage of movement novel	C_2_	***0*.*385***	*-0*.*236*	*0*.*267*
Handling test	Index of defensive behaviour	A	0.196	**0.439**	0.056
		B	0.093	**0.500**	**0.389**
		C_1_	-0.002	**0.402**	**0.420**
		C_2_	-0.064	**0.579**	0.286
		D	-0.150	**0.704**	0.042
		E	-0.268	**0.328**	-0.148
Reactivity test	Movement	A	*-0*.*126*	***-0*.*322***	*-0*.*210*
		C_1_	***-0*.*366***	*0*.*118*	*-0*.*110*
		D	***-0*.*660***	*-0*.*082*	*-0*.*117*
	Tongue flicking	A	0.108	**0.429**	0.285
		C_1_	-0.214	0.029	**-0.376**
		D	-0.244	0.075	**-0.538**
Restraint test	Occurrence of defensive behaviour	A	*0*.*024*	*0*.*086*	*0*.*127*
		C_1_	*-0*.*077*	*0*.*008*	*0*.*151*
		D	*0*.*065*	***0*.*395***	*0*.*222*
		E	*-0*.*241*	***0*.*496***	*-0*.*115*
		F	*-0*.*275*	***0*.*577***	*-0*.*167*
Stress response test	Heart rate stress response 1	E	0.026	**-0.591**	0.076
	Heart rate stress response 2	E	0.157	**-0.540**	0.041
	Breath rate stress response 1	E	0.007	**0.474**	-0.108
	Breath rate stress response 2	E	0.039	**0.449**	-0.313

Summary of the factor loadings from FA in particular life stages A (0–0.5 years), B (0.5–1 years), C_1_ (1–1.5 years), C_2_ (1.5–2 years), D (2–3 years), E (3–4 years) and F (>4 years). Factor 1–3 are factor loadings from FA using the principal axis method, varimax rotation. Bold typeface indicates the factor loadings higher than 0.3, italic typeface indicates the variables that were not statistically repeatable.

Here, we summarise how the behaviours from particular life stages contributed to each of the factors derived from a factor analysis across all tests and life stages (see Table 4). In juveniles (A, B), the first factor of the overall analysis correlated only with the speed of hunting measured in the Feeding trials (Index of catch latency, for life stage (A), r = 0.45). The other variables from the Feeding trials or other behavioural tests were not significantly correlated with this axis. The reason is that multivariate axis here represents the foraging/feeding behavioural profile, which is established in latter stages of development. The second factor of overall analysis (agonistic behavioural profile) was in juveniles mainly correlated with Index of defensive behaviour (r = -0.47). This factor correlated further not only with Movement (r = -0.32), but also with the Tongue flicking (r = 0.43), both measured in the Reactivity test. Not surprisingly, the Tongue flicking is typically correlated with exploratory behaviours in subadult stage (see below and Table 4).

In older juveniles (C_1_, up to 1.5 year; C_2_, up to 2 years), we conducted the same tests as in the previous stage, but with additional tests (e.g., Open field and Activity tests). The correlational structure typical for this stage (see partial PCA analysis) became similar to overall correlational structure calculated here across all the tests and life stages. Interestingly, one behaviour measured in the Feeding trials contributed highly to the first factor (Prey acceptance, for older juveniles (C_1_), r = -0.51) in this stage, which we interpret as a foraging/feeding behavioural profile. Interestingly, the correlation of the Index of defensive behaviour (Handling test) increases loadings with the second factor (for older juveniles (C_1_), r = -0.57; (C_2_), r = -0.59), which we interpret as an agonistic behavioural profile (see above for partial PCA analysis, compare with Table 4)

In subadults (D up to 3 years, E up to 4 years) and adults (F more than 4 years), the overall correlational structure was already stable and very similar to those revealed by a partial PCA describing the correlation across contexts in particular life stages (see above for a partial PCA analysis, compare with Table 4).

**Fig 6:**
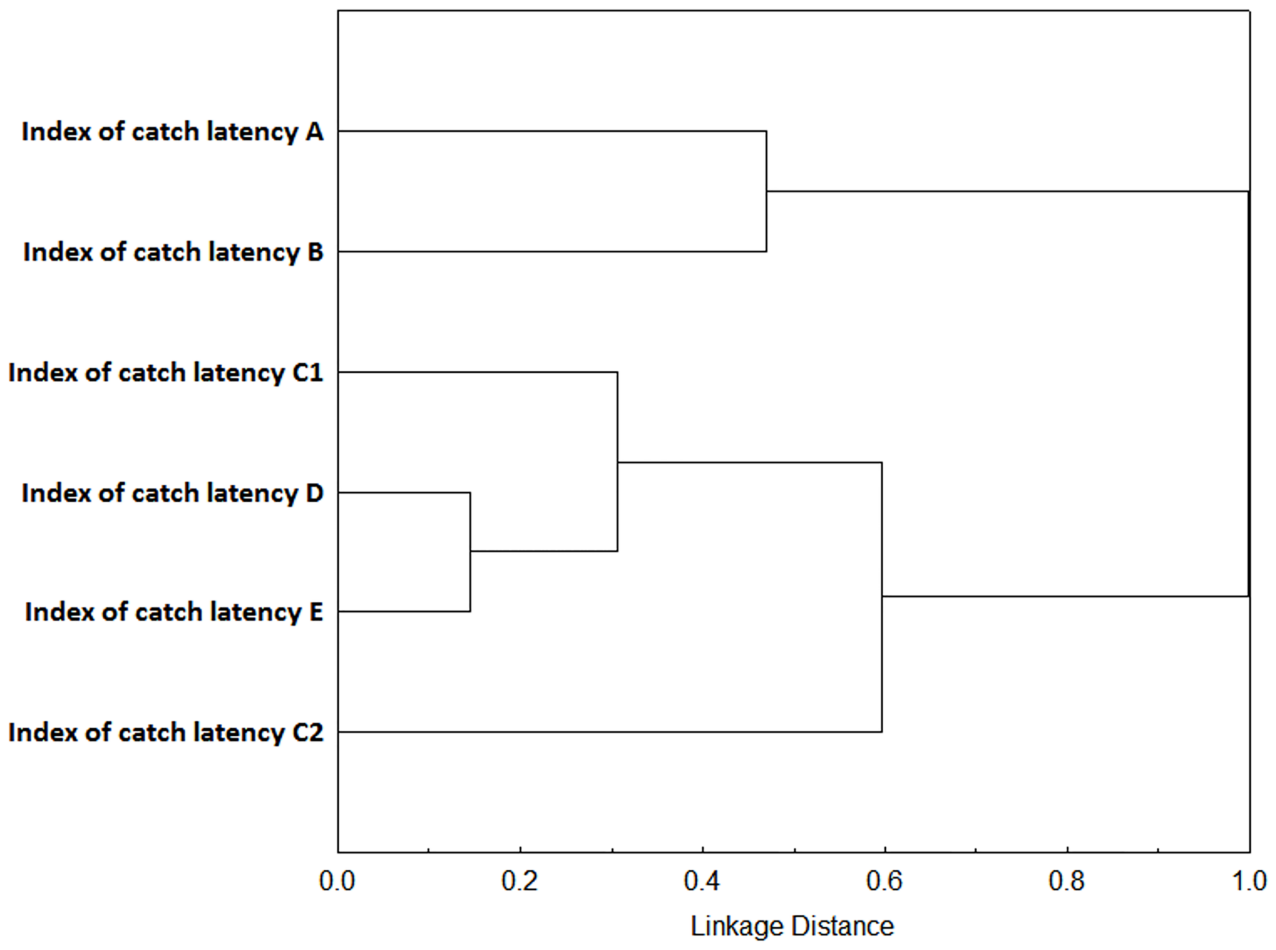
Cluster analysis of variables contributing to Factor 1 (depicting the development of feeding personality). Visualization of the correlation structure by Cluster analysis (1-Pearsons r was selected as metrics and Ward’s method for clustering). The variables that contributed mainly to the first factor in the overall factor analysis were inserted (Index of catch latency). The difference between two parts of life in the feeding context (development over the ontogeny) are clearly visible. The first two life stages (A and B) are clustering together, while the next stages (C_1_, C_2_, D and E) comprise the other cluster.

**Fig 7:**
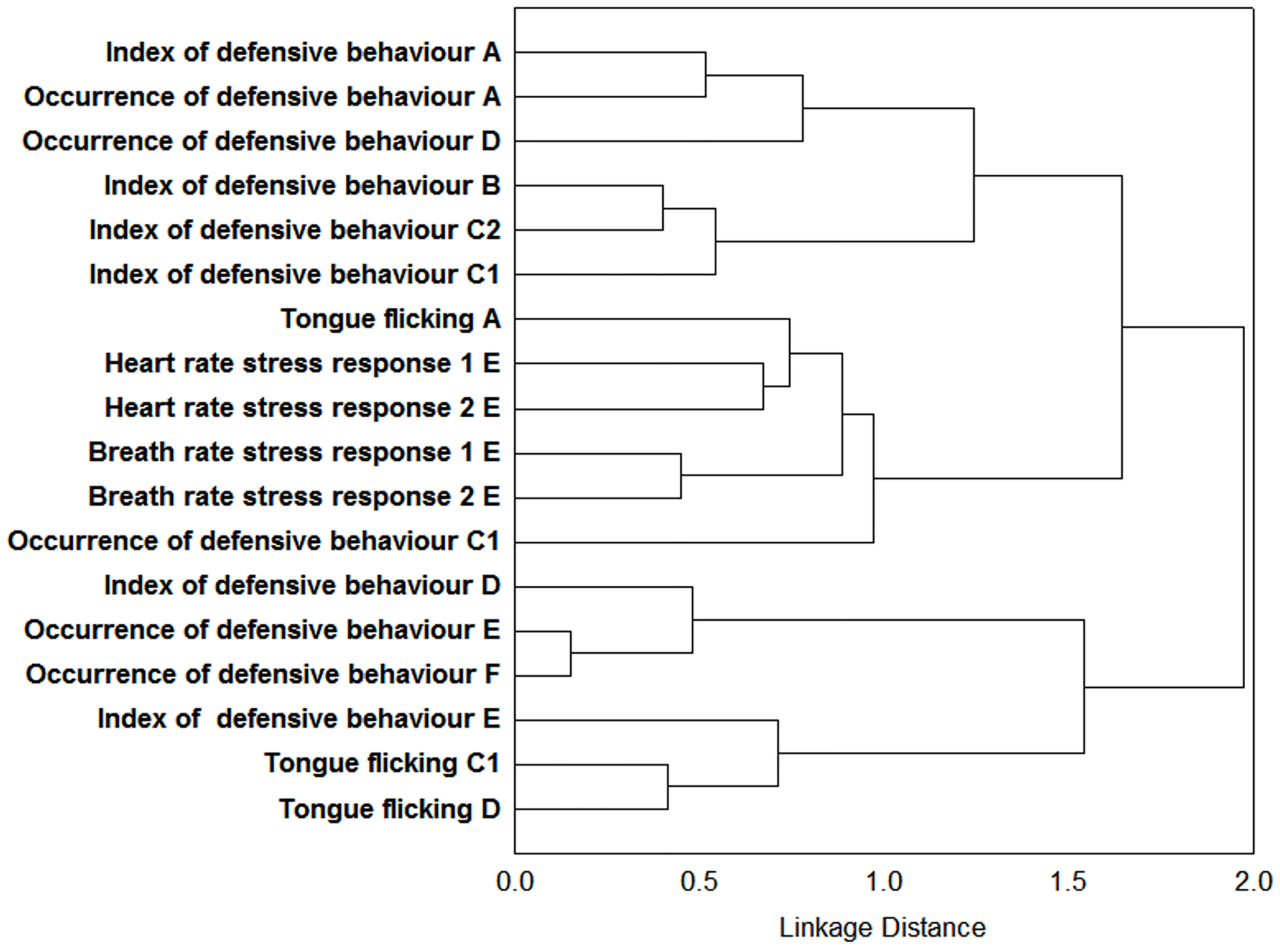
Cluster analysis of variables contributing to Factor 2 (depicting the development of agonistic variables). Visualization of the correlation structure by Cluster analysis (1-Pearsons r was selected as metrics and Ward’s method for clustering). The variables that contributed mainly to the second factor in the overall factor analysis were inserted (most of the variables from the agonistic context and some from the context of exploration). Notice the clustering of agonistic variables from the first life stages (A, B, C_1_ and C_2_) delimiting from the second cluster of agonistic variables (the life stages D and E). Such pattern indicates development of this behaviour over the ontogeny.

**Fig 8:**
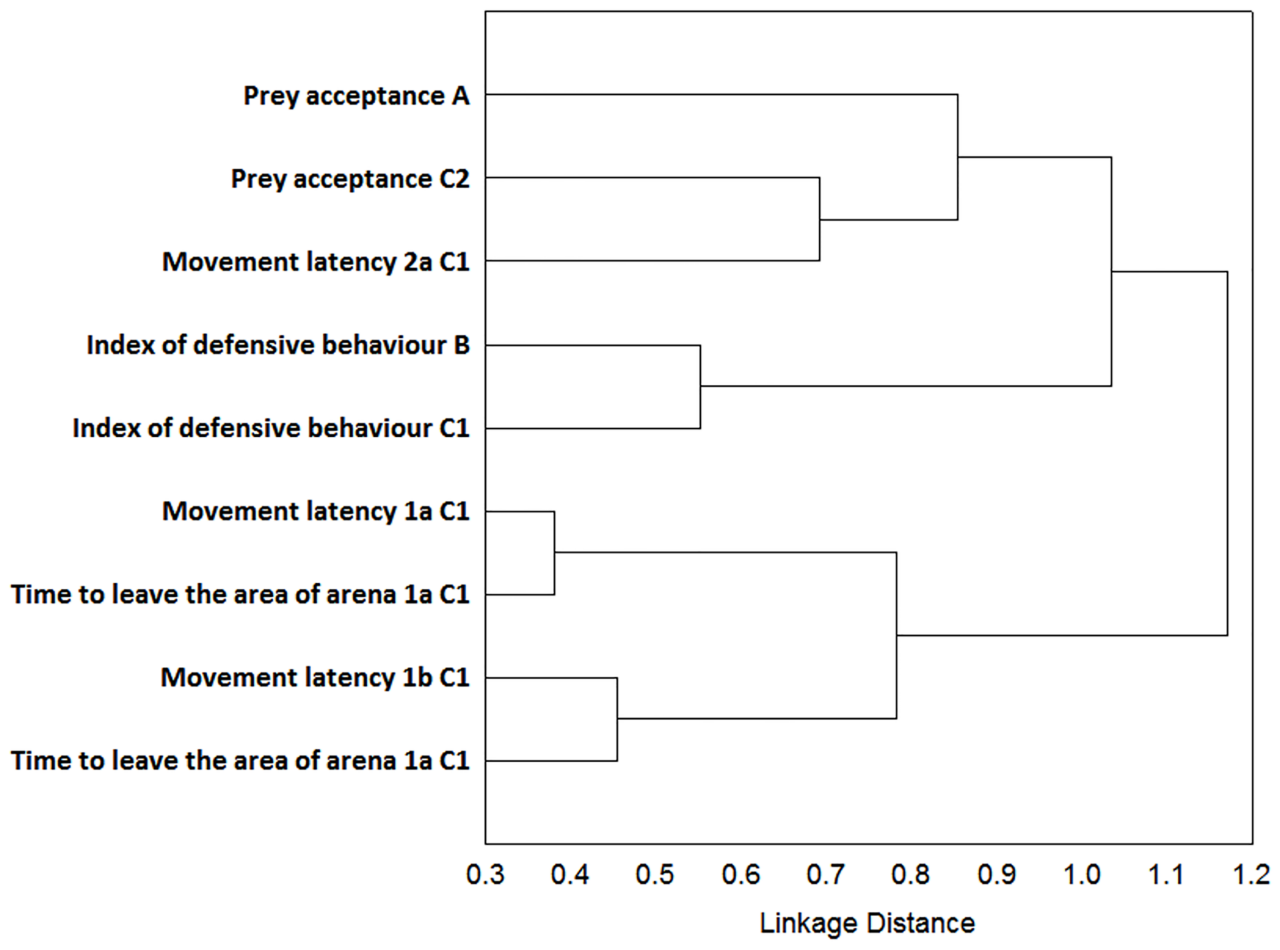
Cluster analysis depicting mixture of variables contributing to the Factor 3. Visualization of the correlation structure by Cluster analysis (1-Pearsons r was selected as metrics and Ward’s method for clustering). The variables that contributed mainly to the third factor in the overall factor analysis were inserted (most of the variables from the context of exploration and some of the agonistic and feeding contexts). Notice the mixture of variables contributing to Factor 3.

To identify the classification structure (depicting structural consistency) caused by different variables measured in different tests across various life stages (A—E) and across all the tests included, we performed a cluster analysis for each of the multivariate axes. The variables that contributed mainly to the first (Factor 1), second (Factor 2), and third (Factor 3) factor in the overall factor analysis (described above; see also Table 4) were inserted into three separate cluster analyses (Figs 6–8). We tried to avoid the variables that were not repeatable or stable and simultaneously without a detected developmental effect. We applied a Cluster Analysis to visualize the correlation structure of our dataset across different life stages. The”1-Pearsons r” was selected as metrics (distance measure) and Ward’s method as a clustering method for Cluster Analysis.

### Body weight as a proximate factor involving personality: Weight as a proxy for overall correlational structure

We correlated the snakes’ body weight at birth and at four years of age with individual factor scores for Factor 1, Factor 2, and Factor 3 extracted from the overall analysis across all the tests and life stages in further analysis. Scores for Factor 1, representing the foraging/feeding behavioural profile was not correlated either with the initial body weight of new-borns nor with the weight at the age of four years (adults). The same was true for a correlation of both body weights and scores for Factor 2, representing the agonistic behavioural profile. The body weight at four years of age but not the one at birth was significantly correlated with individual scores for Factor 3 (Pearson’s r = 0.54, p = 0.002), representing activity-exploratory behavioural profile.

## Discussion

In reptiles, a strong influence of proximate factors, such as genetics [84] or incubation temperature [9, 65, 107] on the personality type may be expected. Proximate factors and/or selection pressure acting early in development modulate behavioural traits that remain stable with respect to both aspects of personality, i.e. differential and structural consistency, to the adulthood. It means that it is not only the particular behaviours, but also the relationship between behavioural traits that remain consistent since the initial life stage. However, a significant difference between selection pressures on juveniles and adults can result in uncoupling the different personality axes [37] to adapt a personality structure to the particular stage of life history. This assumption is in concordance with models predicting that positive feedback stabilizes initial differences in some states (size, energy reserves, condition) and may result in differences in personality, e.g. boldness in foraging context [108, 109]. If the animal changes in some of these states during its development, we can expect personality changes as well.

### Is there an ontogenetic change in coupling of personality traits, implicating different selection pressures in juveniles and adults?

We found an ontogenetic change in feeding/foraging personality as well as agonistic behaviour. Generally, agonistic behaviour and feeding/foraging personality in juveniles were loading on separate axes, and therefore clearly differentiated. During remodelling of personality in subadults, these two behaviours loaded on the same axes. Interestingly, PCA results from the later life stages suggest that these two behaviours form separate axes in adulthood again and the structure is very similar to juvenile personality. Changes in behavioural structure have also been found in zebra finches (*Taeniopygia guttata* [44]). A study conducted on nine-spined sticklebacks (*Pungitius pungitius*) focusing on feeding personality found a decrease in feeding activity during ontogeny, which could have been caused by a selection pressure on fast growth in early development. In an environment with a predation threat, the fish needed to grow faster to avoid predation [110]. Similarly, squids in a feeding context were bolder when juveniles. However, there was an opposite boldness trend in a simulated threat context, i.e. the juveniles were shyer than adults, which points to a similar selection pressure driving the fast growth in both studied species [41, 110]. Our study found principally the same pattern of personality change as was found in squids. In the Northern common boa, boldness in a feeding context and a reaction to a simulated predator threat do not correlate. We found a period of personality restructuring during maturation, as was shown in squids [41]. However, this is not always the case, as some studies document an unchanged structure of behavioural syndrome across ontogeny [35, 40, 45].

The feeding personality is formed mainly by two inter-correlated traits, which are subject to ontogenetic changes. Generally, the speed of hunting positively correlates with subsequent acceptance of familiar prey across the whole ontogeny. In juveniles, it also correlates with acceptance of novel prey. It means that individuals, who immediately decide to catch the prey, are also more willing to accept the offered prey. The process of digestion is very specific in boid snakes. The acceptance of unknown prey is even more risky than acceptance of familiar prey and thus possesses an aspect of boldness. Interestingly, Herzog and Burghardt [76] also found a correlation between latency to move in a novel situation and striking behaviour in one of the three tested species of Garter snakes (*T*. *sirtalis*). In Garter snakes, the speed of decision (e.g. to start moving) and boldness proved to be important and inter-correlated aspects of snake personality. Therefore, some behaviours reflecting boldness in a novel situation and an immediate foraging decision are probably prominent features of snake personality.

The agonistic behaviour forms a second complex of traits. We measured agonistic behaviour in differently designed tests simulating a predator attack with different levels of threat. In juveniles and adults, we found a positive correlation between defensive behaviour regularly measured in the Handling and the Restraint tests. However, we did not detect any coupling of these traits in subadults when the correlations between traits diminished or changed substantially. We found that behaviour intensity in the Reactivity test (when the snake was poked with a rounded tip stick) does not correlate with the other two tests of agonistic behaviour. Moreover, the function of behavioural traits measured in this test changes after the juvenile life stage. Occurrence of movement and tongue flicking in juveniles has negative associations, meaning that the animal is probably either inactive or trying to assess the threatening situation, using olfactory exploration. In subadults, movement occurrence is associated with catch latencies from the feeding trials. Subadults with low speed of hunting are less active in the Reactivity test, therefore the activity/inactivity mode in this test is influenced by the decision speed.

The third source of variability concerns the exploration context, where adults were more active during the activity tests as well as during the exploration ones, sometimes associated also with olfactory and chemical exploration (tongue flicking). However, as we conducted majority of these tests at one life stage, and with a limited number of tests and repetitions, this behavioural type is not significant in our dataset, but needs more precise testing in the future. Interestingly, Chiszar and his colleagues [94] found that there were two contexts in which Plains garter snakes (*Thamnophis radix*) usually increased the frequency of tongue flicking, i.e. typically immediately after handling, when placed in a new arena or presented with new objects [94]. In our Northern common boas, we observed the same pattern of change in tongue flicking frequency. This suggests that tongue flicking in snakes is a part of both the antipredator (in juveniles) and exploratory behaviour (in adults).

### Is personality in the Northern common boa analogous to personality types found in other taxa?

We found that the speed of decision-making constitutes one part of the behavioural syndrome in boas and influences the activity in a simulated threat situation (Reactivity test), as well as feeding personality (especially the speed of hunting). It would be tempting to use the existing terminology of personality studies and call this behavioural syndrome fast vs slow snakes. The fast/slow personality type was found in multiple species (*Parus major* [111]; *Corvus corax* [112]; *Carduelis chloris* [113]; *Tamias striatus* [114]; *Amatitlania nigrofasciata* [115]). It was often associated, similarly to our results, with aggression [116] and boldness [117]. However, the fast/slow personality type is based on exploratory behaviour, e.g. in great tits (*Parus major* [111]), where birds with the fast personality type explored the novel environment quickly, but superficially, while the “slow” birds explored more thoroughly, but slowly. In our study, the exploratory behaviour did not correlate with the speed of decision-making. Thus, using the term “fast” and “slow” personality type for boas would be slightly misleading.

Another part of the behavioural syndrome in boas is constituted of agonistic behaviour in the Handling test and the Restraint tests and their heart and breath rate response in the Stress response test. The individual variability in reaction to a stressful situation was described in rodents as a “coping style”, where the passive, non-aggressive animals were called “reactive” and the more aggressive animals actively avoiding the stressor were labelled as “proactive” [118]. This behavioural syndrome is also associated with behavioural flexibility and physiological parameters [119]. We observed some aspects of this behavioural syndrome in our subjects, but we found no association with behavioural flexibility (reaction to novel stimuli) in novel prey tests.

### Does the slow life-style influence personality traits?

We found that the growth rate (expressed as the body weight at four years of age) significantly correlates with individual scores for activity-exploratory behaviour, but not with feeding and agonistic behavioural profile. It suggests that performance tests concerning activity and exploration are still appropriate as in other species of reptiles [24, 32, 74] although boid snakes are sit-and-wait predators [80]. We also found a close relationship between the heart and breath rate (representing a resting and stressful indicator of metabolism) and agonistic behaviour across life-stages (see Fig 7). The physiological stress response in the Northern common boa revealed an increased heart rate, but a counterintuitive breath rate decrease in an antipredatory context (see Table 4). The breath rate is usually increased under stressful situations [120–123], nevertheless snake physiology is quite specific in regards to oxygen consumption. Snakes are able to sustain quite a long time without oxygen due to their specific prey intake [124]. Nevertheless, a large heart rate stress response is not connected with peaceful, more reactive personality (e.g. in Handling test).

### Are personality traits in snakes individually consistent across development?

Our proposed hypothesis states that if the selection pressures in juveniles and adults differ, the level of consistency during development will differ as well. We examined the effect of development on behaviour in different contexts (feeding, agonistic etc.) and repeatability across all life stages. We expected the variables best reflecting stable personality without development to be repeatable and have good concordance, but to not change during ontogeny. In our data, we found no variable matching these predictions. Despite the fact that some variables are affected by development, they should still show good concordance and repeatability to be considered as a personality trait. Stamps and Krishnan [125] postulate that personality traits measured in later life stages should be more consistent, which might cause worse repeatability in variables measured continuously from the first year of life.

In the feeding context, the proportion of accepted prey and speed of hunting were both significantly different in juveniles. However, their repeatability and concordance were significant across development. We can expect a consistent selection pressure during development resulting in changes of the mean level of behaviour in late juvenile/ early subadult life stages. We found a slight improvement in consistency of the hunting speed during development of feeding personality, but a decrease in consistency of prey acceptance. The boldness in the novel prey context did not show significant repeatability, but all the variables had significant concordance. This suggests that there is some development of boldness, resulting in a change of the mean level of the variables, but not in the animals’ rank order.

The Capture success of novel prey changes during development and has good concordance, but is not repeatable. We explain this pattern by this behavioural trait’s complicated development, which is not stable, but the animals rank order tend to be consistent. The type of prey (live or dead) can also influence the consistency, as well as novelty of the prey for the snakes. The novel prey tests should be designed more carefully in the future with respect to the novel prey category, e.g. to separate live and dead prey items. It was demonstrated that juvenile western yellow-bellied racers (*Coluber constrictor mormon*) as well accepted only 60% of offered dead prey items with some individuals that refused to accept the dead prey completely. Other snakes needed much longer to catch the dead prey compared to a live one [90]. Therefore, we can assume that the overall mean boldness is different in the adult life stage, but the individual relative rank order remains unchanged throughout development. This makes the levels of boldness a candidate personality trait after a careful consideration of its dynamic.

The mean level of agonistic behaviour in any of the tests did not change across development. Defensive behaviour in the Restraint test showed poor concordance and non-significant repeatability, which suggests that the snakes behaved randomly. It should be due to a small number of test repetitions, because one measurement of one/ zero activity expressed by a snake here (defensive or calm response) is influenced by many internal as well as external factors. This test was administered also in very long inter-test intervals (months) that lowered repeatability of personality traits [38, 39].

Originally, the occurrence of Movement (measured in the Reactivity test) had no significant concordance, nor repeatability. According to predictions of Stamps and Krishnan [125], behavioural consistency should improve during the development. This prediction is valid for the occurrence of Movement in the Reactivity test. When we calculated the Kendall’s coefficient of concordance only for subadults and adults, the coefficient of concordance improved greatly and was significant. It suggests a change of this behaviour after the first year of life, which might cause the overall poor repeatability, but is not systematic enough to make a significant life stage effect. We explain this by consistent inter-individual differences stabilizing after the first year of life.

In the Tongue flicking (measured in the Reactivity test) we found no developmental change, and poor concordance, but good repeatability. From our experience, we know that most of the individuals are highly consistent, but a couple of them behave inconsistently. These few inconsistent individuals might have caused the poor concordance without influencing the overall repeatability. The same explanation can be applied to the Index of defensive behaviour (measured in the Handling test). In colubrid snakes from the genus *Thamnophis*, good repeatability of strike scores (the ordinal variable combining the intensity of defensive reaction, body posture, and the number of strikes) was found in tests simulating a predatory attack. Repeatability between tests conducted in two consecutive days [76] was for juveniles of *T*. *melanogaster* (r = 0.64), for *T*. *sirtalis* (r = 0.56), for *T*. *butleri* (r = 0.76), and for adult *T*. *melanogaster* females (r = 0.84). Our repeatability was lower (r = 0.39), but still significant. This difference might have been caused by different methods or inter-specific differences.

Although the structure of behaviour in the tests of agonistic behaviour changes, repeatability of defensive behaviour in the Handling test is significant, as well as repeatability of Tongue flicking in the Reactivity test. Movement in the Reactivity test is also affected by the structural change, however after the first year of life it has a good concordance. Overall, the effect of development on agonistic behaviour is smaller than the effect on feeding personality.

The exploratory behaviour in the open field was measured only in one life stage (C), but within the life stage it had good repeatability and rather good concordance. The time to leave the arena is a better candidate for a personality trait as it has better concordance than Movement latency. The effect of development of this personality trait requires more testing.

Our data on repeatability generally support the hypothesis proposed by Bell and her colleagues [2] that differences between traits involving temporal consistency (e.g. differential consistency) arises from differences in their underlying ecological importance. In our data, the best repeatability was found in variables measured in tests designed to cover foraging and feeding contexts and some variables measuring an individual defensive response during handling.

### Does the personality remain stable during development? How do proximate and ultimate factors influence establishment of personality?

We discovered a correlation between principally different personality tests mainly in the foraging and antipredatory context, which suggests the existence of foraging/feeding and agonistic personality. As most of the personality tests were performed throughout ontogeny from birth to sexual maturity we detected the influence of development on structural and differential consistency of personality.

If we also look at the stability of behavioural profiles over time (context generality), those concerning aspects of feeding and defensive behaviour are both presented in juveniles as well as adults. However, both of them restructure during the subadult life stage. For juveniles, the set of traits forming the complex defensive response is important, while in subadults and adults the features associated with foraging and feeding behaviours are those shaping the snake’s personality. However, the mean level of variables reflecting feeding personality also changes with development. The juveniles are always different from adults and, in many aspects also from subadults. Therefore, we conclude that the feeding personality changes after the juvenile life stage and remains mostly consistent throughout the rest of the development.

The developmental change in agonistic behaviour is structural, but does not affect the mean level of behaviour. There might be slightly different selection pressures in juvenile and adult life stages, especially on behaviour in a low-level threat situation, however these two life stages are mostly similar in the personality structure. There is a period of structural change of personality during the second year of life. In this life stage (subadult) the snakes are not so vulnerable to predators and it is possible that they change their perception as to what constitutes a threat for them. Apart from body growth, they also start investing in sexual maturation, which may cause the change in personality structure. However, to answer this question a more detailed study of life history traits during personality remodelling is needed.

The changes of personality during the subadult life stage is supported by some empirical studies ([41, 126, 127] but see [77]), but does not correspond with any theoretical model.

In regards to proximate factors affecting personality of the Northern common boa, they are probably associated with the agonistic behavioural profile. Although we measured them as physiological parameters (heart and breath rate stress response) in adults only, they even cluster with variables concerning the agonistic behavioural profile across most of the life stages. Individuals with a high score of Prey acceptance are characterized by fast decision-making, are more aggressive (often bites when disturbed) and in terms of physiological parameters are more prone to be stressed (high heart rate and low breath rate). Because the stress response measured in adult age is associated with behaviour in juvenile life-stage (see Fig 7), it should be considered not only as a marker, but also as a proximate factor, influencing the development of personality in snakes. The complicated role of stress in snake personality should be subjected to further experiments. Moreover, we found no relationship between juvenile/adult body size and personality concerning the feeding/foraging and agonistic behavioural profile. Thus, we propose to explore another concept of proximate factors influencing the establishment of personality in boas concerning the body size. It is the body growth with a detail examination of growth increments across ontogeny (especially during the period of sexual maturation and following growth attenuation) which could bring a new insight into the personality development in snakes.

## Supporting information

S1 File List of variables. The list of contexts, tests and variables measured in our manuscript. More detailed definitions of these variables are provided.
(DOC)

S1 Table Table of Kendal's coefficients. Kendall's coefficients of concordance for each of the variables, reflecting the rank order consistency of the variable. The numbers in brackets represent data after we excluded the first year of life. ** Asterisks are indicating the statistical significance of Kendal's coefficient of concordance W (P < 0.001); * P < 0.01.
(XLS)

S2 Table Table of dependent variables. Table of variables used for statistical analyses of repeatability, principal component analysis, factor analysis and correlation analyses. Data are transformed prior the normal distribution. For specific transformation and explanation of the variables see Table 1. Variables Breath rate stress response 1 and 2 were transformed for repeatability test by adding the constant to improve the distribution of data.
(XLS)

S3 Table Table of factor scores. Table of identity, sex, initial (A) and final (F) body weight (in grams) and factor scores from principal component analyses (for selected life stages A, C1, D and E) as well as from factor analyses including the whole dataset of dependent variables across all life stages.
(XLS)

## References

[1] Dall SRX, Houston AI, McNamara JM. The behavioural ecology of personality: consistent individual differences from an adaptive perspective. Ecology Letters. 2004;7(8):734–9. 10.1111/j.1461-0248.2004.00618.x

[2] Bell AM, Hankison SJ, Laskowski KL. The repeatability of behaviour: a meta-analysis. Animal Behaviour. 2009;77(4):771–83. 10.1016/j.anbehav.2008.12.022 . 24707058 PMC3972767

[3] Stamps J, Groothuis TGG. The development of animal personality: relevance, concepts and perspectives. Biological Reviews. 2010;85(2):301–25. 10.1111/j.1469-185X.2009.00103.x . 19961473

[4] Réale D, Reader SM, Sol D, McDougall PT, Dingemanse NJ. Integrating animal temperament within ecology and evolution. Biological Reviews. 2007;82(2):291–318. 10.1111/j.1469-185X.2007.00010.x . 17437562

[5] Gosling SD. From mice to men: What can we learn about personality from animal research? Psychological Bulletin. 2001;127(1):45–86. 10.1037//0033-2909.127.1.45 . 11271756

[6] Sih A, Bell AM, Johnson JC, Ziemba RE. Behavioral syndromes: An integrative overview. Quarterly Review of Biology. 2004;79(3):241–77. 10.1086/422893 . 15529965

[7] Réale D, Dingemanse N. Animal Personality: eLS.; 2012.

[8] Cote J, Clobert J. Social personalities influence natal dispersal in a lizard. Proceedings of the Royal Society B-Biological Sciences. 2007;274(1608):383–90. 10.1098/rspb.2006.3734 PMC1702372 17164202

[9] Li H, Holleley CE, Elphick M, Georges A, Shine R. The behavioural consequences of sex reversal in dragons. Proceedings of the Royal Society B-Biological Sciences. 2016;283(1832) 10.1098/rspb.2016.0217

[10] Magnhagen C. Social influence on the correlation between behaviours in young-of-the-year perch. Behavioral Ecology and Sociobiology. 2007;61(4):525–31. 10.1007/s00265-006-0280-3

[11] Zhao DP, Feng PS. Temperature increase impacts personality traits in aquatic non-native species: Implications for biological invasion under climate change. Current Zoology. 2015;61(6):966–71 10.1093/czoolo/61.6.966 PMC7098674 32256532

[12] Snijders L, van Rooij EP, Henskens MFA, van Oers K, Naguib M. Dawn song predicts behaviour during territory conflicts in personality-typed great tits. Animal Behaviour. 2015;109:45–52. 10.1016/j.anbehav.2015.07.037

[13] Miller R, Laskowski KL, Schiestl M, Bugnyar T, Schwab C. Socially Driven Consistent Behavioural Differences during Development in Common Ravens and Carrion Crows. Plos One. 2016;11(2) 10.1371/journal.pone.0148822 PMC4746062 26848954

[14] Sommer-Trembo C, Zimmer C, Jourdan J, Bierbach D, Plath M. Predator experience homogenizes consistent individual differences in predator avoidance. Journal of Ethology. 2016;34(2):155–65. 10.1007/s10164-016-0460-1

[15] Vetter SG, Brandstatter C, Macheiner M, Suchentrunk F, Gerritsmann H, Bieber C. Shy is sometimes better: personality and juvenile body mass affect adult reproductive success in wild boars, *Sus scrofa*. Animal Behaviour. 2016;115:193–205. 10.1016/j.anbehav.2016.03.026

[16] Sinn DL, Moltschaniwskyj NA. Personality traits in dumpling squid (*Euprymna tasmanica*): Context-specific traits and their correlation with biological characteristics. Journal of Comparative Psychology. 2005;119(1):99–110. 10.1037/0735-7036.119.1.99 . 15740434

[17] Kralj-Fišer S, Schuett W. Studying personality variation in invertebrates: why bother? Animal Behaviour. 2014;91:41–52. 10.1016/j.anbehav.2014.02.016

[18] Carere C, Drent PJ, Privitera L, Koolhaas JM, Groothuis TGG. Personalities in great tits, *Parus major*: stability and consistency. Animal Behaviour. 2005;70:795–805. 10.1016/j.anbehav.2005.01.003

[19] Aplin LM, Farine DR, Mann RP, Sheldon BC. Individual-level personality influences social foraging and collective behaviour in wild birds. Proceedings of the Royal Society B-Biological Sciences. 2014;281(1789) 10.1098/rspb.2014.1016 PMC4100518 24990682

[20] Bousquet CAH, Petit O, Arrive M, Robin JP, Sueur C. Personality tests predict responses to a spatial-learning task in mallards, *Anas platyrhynchos*. Animal Behaviour. 2015;110:145–54. 10.1016/j.anbehav.2015.09.024

[21] McCowan LSC, Mainwaring MC, Prior NH, Griffith SC. Personality in the wild zebra finch: exploration, sociality, and reproduction. Behavioral Ecology. 2015;26(3):735–46. 10.1093/beheco/aru239

[22] Class B, Brommer JE. Senescence of personality in a wild bird. Behavioral Ecology and Sociobiology. 2016;70(5):733–44. 10.1007/s00265-016-2096-0

[23] Réale D, Martin J, Coltman DW, Poissant J, Festa-Bianchet M. Male personality, life-history strategies and reproductive success in a promiscuous mammal. Journal of Evolutionary Biology. 2009;22(8):1599–607. 10.1111/j.1420-9101.2009.01781.x . 19555442

[24] Careau V, Garland T. Performance, Personality, and Energetics: Correlation, Causation, and Mechanism. Physiological and Biochemical Zoology. 2012;85(6):543–71. 10.1086/666970 . 23099454

[25] Montiglio PO, Garant D, Pelletier F, Réale D. Personality differences are related to long-term stress reactivity in a population of wild eastern chipmunks, *Tamias striatus*. Animal Behaviour. 2012;84(4):1071–9. 10.1016/j.anbehav.2012.08.010

[26] Briard L, Dorn C, Petit O. Personality and Affinities Play a Key Role in the Organisation of Collective Movements in a Group of Domestic Horses. Ethology. 2015;121(9):888–902. 10.1111/eth.12402

[27] Sih A, Kats LB, Maurer EF. Behavioural correlations across situations and the evolution of antipredator behaviour in a sunfish-salamander system. Animal Behaviour. 2003;65:29–44. 10.1006/anbe.2002.2025

[28] Wilson ADM, Krause J. Personality and metamorphosis: is behavioral variation consistent across ontogenetic niche shifts? Behavioral Ecology. 2012;23(6):1316–23. 10.1093/beheco/ars123

[29] Urszan TJ, Torok J, Hettyey A, Garamszegi LZ, Herczeg G. Behavioural consistency and life history of *Rana dalmatina* tadpoles. Oecologia. 2015;178(1):129–40. 10.1007/s00442-014-3207-0 . 25656582

[30] Carazo P, Noble DWA, Chandrasoma D, Whiting MJ. Sex and boldness explain individual differences in spatial learning in a lizard. Proceedings of the Royal Society B-Biological Sciences. 2014;281(1782) 10.1098/rspb.2013.3275 PMC3973267 24619443

[31] McEvoy J, While GM, Sinn DL, Carver S, Wapstra E. Behavioural syndromes and structural and temporal consistency of behavioural traits in a social lizard. Journal of Zoology. 2015;296(1):58–66. 10.1111/jzo.12217

[32] Mell H, Josserand R, Decenciere B, Artacho P, Meylan S, Le Galliard JF. Do personalities co-vary with metabolic expenditure and glucocorticoid stress response in adult lizards? Behavioral Ecology and Sociobiology. 2016;70(6):951–61. 10.1007/s00265-016-2117-z

[33] Bergmüller R, Taborsky M. Animal personality due to social niche specialisation. Trends in Ecology & Evolution. 2010;25(9):504–11. 10.1016/j.tree.2010.06.012 20638151

[34] Stamps JA, Biro PA. Personality and individual differences in plasticity. Current Opinion in Behavioral Sciences. 2016;12:18–23. 10.1016/j.cobeha.2016.08.008

[35] Müller T, Müller C. Behavioural phenotypes over the lifetime of a holometabolous insect. Frontiers in Zoology. 2015;12 10.1186/1742-9994-12-s1-s8 PMC4722364 26816525

[36] Guenther A, Trillmich F. Photoperiod influences the behavioral and physiological phenotype during ontogeny. Behavioral Ecology. 2013;24(2):402–11. 10.1093/beheco/ars177

[37] Groothuis TGG, Trillmich F. Unfolding Personalities: The Importance of Studying Ontogeny. Developmental Psychobiology. 2011;53(6):641–55. 10.1002/dev.20574 . 21866544

[38] Lessells CM, Boag PT. Unrepeatable repeatabilities—A Common mistake. Auk. 1987;104(1):116–21

[39] Nakagawa S, Schielzeth H. Repeatability for Gaussian and non-Gaussian data: a practical guide for biologists. Biological Reviews. 2010;85(4):935–56. 10.1111/j.1469-185X.2010.00141.x . 20569253

[40] Bell AM, Stamps JA. Development of behavioural differences between individuals and populations of sticklebacks, *Gasterosteus aculeatus*. Animal Behaviour. 2004;68:1339–48. 10.1016/j.anbehav.2004.05.007

[41] Sinn DL, Gosling SD, Moltschaniwskyj NA. Development of shy/bold behaviour in squid: context-specific phenotypes associated with developmental plasticity. Animal Behaviour. 2008;75:433–42. 10.1016/j.anbehav.2007.05.008

[42] Dingemanse NJ, Both C, Drent PJ, Van Oers K, Van Noordwijk AJ. Repeatability and heritability of exploratory behaviour in great tits from the wild. Animal Behaviour. 2002;64:929–38. 10.1006/anbe.2002.2006

[43] Weiss A, King JE, Hopkins WD. A cross-setting study of chimpanzee (*Pan troglodytes*) personality structure and development: Zoological parks and Yerkes National Primate Research Center. American Journal of Primatology. 2007;69(11):1264–77. 10.1002/ajp.20428 . 17397036 PMC2654334

[44] Wuerz Y, Krueger O. Personality over ontogeny in zebra finches: long-term repeatable traits but unstable behavioural syndromes. Frontiers in Zoology. 2015;12 10.1186/1742-9994-12-s1-s9 PMC4722341 26813709

[45] Gyuris E, Fero O, Barta Z. Personality traits across ontogeny in firebugs, *Pyrrhocoris apterus*. Animal Behaviour. 2012;84(1):103–9. 10.1016/j.anbehav.2012.04.014

[46] Hedrick AV, Kortet R. Sex differences in the repeatability of boldness over metamorphosis. Behavioral Ecology and Sociobiology. 2012;66(3):407–12. 10.1007/s00265-011-1286-z

[47] Petelle MB, McCoy DE, Alejandro V, Martin JGA, Blumstein DT. Development of boldness and docility in yellow-bellied marmots. Animal Behaviour. 2013;86(6):1147–54. 10.1016/j.anbehav.2013.09.016

[48] Edenbrow M, Croft DP. Environmental and genetic effects shape the development of personality traits in the mangrove killifish *Kryptolebias marmoratus*. Oikos. 2013;122(5):667–81. 10.1111/j.1600-0706.2012.20556.x

[49] Guenther A, Finkemeier MA, Trillmich F. The ontogeny of personality in the wild guinea pig. Animal Behaviour. 2014;90:131–9. 10.1016/j.anbehav.2014.01.032

[50] Boissy A. Fear and fearfulness in animals. Quarterly Review of Biology. 1995;70(2):165–91. 10.1086/418981 . 7610234

[51] Carere C, Caramaschi D, Fawcett TW. Covariation between personalities and individual differences in coping with stress: Converging evidence and hypotheses. Current Zoology. 2010;56(6):728–40

[52] de Boer SF, Buwalda B, Koolhaas JM. Untangling the neurobiology of coping styles in rodents: Towards neural mechanisms underlying individual differences in disease susceptibility. Neuroscience and Biobehavioral Reviews. 2017;74:401–22. 10.1016/j.neubiorev.2016.07.008 . 27402554

[53] Stamps JA, Groothuis TGG. Developmental perspectives on personality: implications for ecological and evolutionary studies of individual differences. Philosophical Transactions of the Royal Society B-Biological Sciences. 2010;365(1560):4029–41. 10.1098/rstb.2010.0218 PMC2992751 21078655

[54] Trillmich F, Hudson R. The Emergence of Personality in Animals: The Need for a Developmental Approach. Developmental Psychobiology. 2011;53(6):505–9. 10.1002/dev.20573 . 21866537

[55] Chapman BB, Hegg A, Ljungberg P. Sex and the Syndrome: Individual and Population Consistency in Behaviour in Rock Pool Prawn *Palaemon elegans*. Plos One. 2013;8(3) 10.1371/journal.pone.0059437 PMC3598797 23555034

[56] Stamps JA, Krishnan VV. Individual differences in the potential and realized developmental plasticity of personality traits. Frontiers in Ecology and Evolution. 2014;2(69) 10.3389/fevo.2014.00069

[57] Smith BR, Blumstein DT. Structural consistency of behavioural syndromes: does predator training lead to multi-contextual behavioural change? Behaviour. 2012;149(2):187–213. 10.1163/156853912x634133

[58] Naguib M, Florcke C, van Oers K. Effects of Social Conditions During Early Development on Stress Response and Personality Traits in Great Tits (*Parus major*). Developmental Psychobiology. 2011;53(6):592–600. 10.1002/dev.20533 . 21365640

[59] Hudson R, Bautista A, Reyes-Meza V, Montor JM, Rodel HG. The Effect of Siblings on Early Development: A Potential Contributor to Personality Differences in Mammals. Developmental Psychobiology. 2011;53(6):564–74. 10.1002/dev.20535 . 21866540

[60] Talarovičová A, Kršková L, Blazeková J. Testosterone enhancement during pregnancy influences the 2D:4D ratio and open field motor activity of rat siblings in adulthood. Hormones and Behavior. 2009;55(1):235–9. 10.1016/j.yhbeh.2008.10.010 . 19022257

[61] Overli O, Sorensen C, Pulman KGT, Pottinger TG, Korzan WJ, Summers CH, et al Evolutionary background for stress-coping styles: Relationships between physiological, behavioral, and cognitive traits in non-mammalian vertebrates. Neuroscience and Biobehavioral Reviews. 2007;31(3):396–412. 10.1016/j.neubiorev.2006.10.006 . 17182101

[62] Mayer M, Shine R, Brown GP. Bigger babies are bolder: effects of body size on personality of hatchling snakes. Behaviour. 2016;153(3):313–23. 10.1163/1568539x-00003343

[63] Besson AA, Lagisz M, Senior AM, Hector KL, Nakagawa S. Effect of maternal diet on offspring coping styles in rodents: a systematic review and meta-analysis. Biological Reviews. 2016;91(4):1065–80. 10.1111/brv.12210 . 26179142

[64] Nyqvist MJ, Gozlan RE, Cucherousset J, Britton JR. Behavioural Syndrome in a Solitary Predator Is Independent of Body Size and Growth Rate. Plos One. 2012;7(2) 10.1371/journal.pone.0031619 PMC3282768 22363687

[65] Trník M, Albrechtová J, Kratochvíl L. Persistent Effect of Incubation Temperature on Stress-Induced Behavior in the Yucatan Banded Gecko (*Coleonyx elegans*). Journal of Comparative Psychology. 2011;125(1):22–30. 10.1037/a0021186 . 21244135

[66] Siviter H, Deeming DC, Rosenberger J, Burman OHP, Moszuti SA, Wilkinson A. The impact of egg incubation temperature on the personality of oviparous reptiles. Animal Cognition. 2017;20(1):109–16. 10.1007/s10071-016-1030-1 . 27599495 PMC5274644

[67] Carter AW, Paitz RT, McGhee KE, Bowden RM. Turtle hatchlings show behavioral types that are robust to developmental manipulations. Physiology & Behavior. 2016;155:46–55. 10.1016/j.physbeh.2015.11.034 26657026

[68] Hawlena D, Boochnik R, Abramsky Z, Bouskila A. Blue tail and striped body: why do lizards change their infant costume when growing up? Behavioral Ecology. 2006;17(6):889–96. 10.1093/beheco/arl023

[69] Wilson D, Heinsohn R, Endler JA. The adaptive significance of ontogenetic colour change in a tropical python. Biology Letters. 2007;3(1):40–3. 10.1098/rsbl.2006.0574 . 17443961 PMC2373822

[70] Landová E, Jančúchová-Lásková J, Musilová V, Kadochová S, Frynta D. Ontogenetic switch between alternative antipredatory strategies in the leopard gecko (*Eublepharis macularius*): defensive threat versus escape. Behavioral Ecology and Sociobiology. 2013;67(7):1113–22. 10.1007/s00265-013-1536-3

[71] Wolf M, van Doorn GS, Leimar O, Weissing FJ. Life-history trade-offs favour the evolution of animal personalities. Nature. 2007;447(7144):581–4. 10.1038/nature05835 . 17538618

[72] Clark CW. Antipredator behavior and the asset-protection principle. Behavioral Ecology. 1994;5(2):159–70. 10.1093/beheco/5.2.159

[73] Frynta D, Vejvodová T, Šimková O. Sex allocation and secondary sex ratio in Cuban boa (*Chilabothrus angulifer*): mother's body size affects the ratio between sons and daughters. Science of Nature. 2016;103(5–6) 10.1007/s00114-016-1369-9 27216175

[74] Le Galliard JF, Paquet M, Cisel M, Montes-Poloni L. Personality and the pace-of-life syndrome: variation and selection on exploration, metabolism and locomotor performances. Functional Ecology. 2013;27(1):136–44. 10.1111/1365-2435.12017

[75] Herzog HA, Burghardt GM. Development of antipredator responses in snakes. III. Stability of individual and litter differences over the first year of life. Ethology. 1988;77(3):250–8

[76] Herzog HA, Burghardt GM. Development of antipredator responses in snakes. I. Defensive and open-field behaviors in newborns and adults of three species of garter snakes (*Thamnophis melanogaster*, *T*. *sirtalis*, *T*. *butleri*). Journal of Comparative Psychology. 1986;100(4):372–9. 10.1037/0735-7036.100.4.372

[77] Caspi A, Roberts BW, Shiner RL. Personality development: Stability and change. Annual Review of Psychology. 2005;56:453–84. 10.1146/annurev.psych.55.090902.141913 . 15709943

[78] Card DC, Schield DR, Adams RH, Corbin AB, Perry BW, Andrew AL, et al Phylogeographic and population genetic analyses reveal multiple species of Boa and independent origins of insular dwarfism. Molecular Phylogenetics and Evolution. 2016;102:104–16. doi: 10.1016/j.ympev.2016.05.034 27241629 PMC5894333

[79] Hynková I, Starostová Z, Frynta D. Mitochondrial DNA Variation Reveals Recent Evolutionary History of Main *Boa constrictor* Clades. Zoological Science. 2009;26(9):623–31. 10.2108/zsj.26.623 . 19799513

[80] Reed R, Rodda G. Giant Constrictors: Biological and Management Profiles and an Establishment Risk Assessment for Nine Large Species of Pythons, Anacondas, and the Boa Constrictor: U.S. Geological Survey Open-File Report; 2009. 302 p.

[81] Gardner-Santana LC, Beaupre SJ. Timber Rattlesnakes (*Crotalus horridus*) Exhibit Elevated and Less Variable Body Temperatures during Pregnancy. Copeia. 2009;(2):363–8. 10.1643/cp-07-271

[82] Schuett GW, Hardy DL, Earley RL, Greene HW. Does prey size induce head skeleton phenotypic plasticity during early ontogeny in the snake Boa constrictor? Journal of Zoology. 2005;267:363–9. 10.1017/s0952836905007624

[83] Lind AJ, Welsh HH. Ontogenetic changes in foraging behavior and habitat use by the Oregon garter snake, *Thamnophis atratus hydrophilus*. Animal Behaviour. 1994;48(6):1261–73. 10.1006/anbe.1994.1362

[84] Arnold SJ, Bennett AF. Behavioral variation in natural populations. III. Antipredator displays in the garter snake *Thamnophis radix*. Animal Behaviour. 1984;32(NOV):1108–18. 10.1016/s0003-3472(84)80227-4

[85] Montgomery GG, Rand AS. Movements, body temperature and hunting strategy of a Boa constrictor. Copeia. 1978;(3):532–3

[86] Webb JK, Brook BW, Shine R. Does foraging mode influence life history traits? A comparative study of growth, maturation and survival of two species of sympatric snakes from south-eastern Australia. Austral Ecology. 2003;28(6):601–10. 10.1046/j.1442-9993.2003.t01-1-01316.x

[87] Herzog HA, Bailey BD. Development of antipredator responses in snakes. II. Effects of recent feeding on defensive behaviors of juvenile garter snakes (*Thamnophis sirtalis*). Journal of Comparative Psychology. 1987;101(4):387–9. 10.1037/0735-7036.101.4.387

[88] Secor SM. Regulation of digestive performance: a proposed adaptive response. Comparative Biochemistry and Physiology a-Molecular and Integrative Physiology. 2001;128(3):565–77 10.1016/s1095-6433(00)00325-1 11246045

[89] Ott BD, Secor SM. Adaptive regulation of digestive performance in the genus *Python*. Journal of Experimental Biology. 2007;210(2):340–56. 10.1242/jeb.02626 17210969

[90] Herzog H, Burghardt G. Prey movement and predatory behavior of juvenile western yellow-bellied racers, *Coluber constrictor mormon*. Herpetologica. 1974:285–9.

[91] Candland DK, Nagy ZM. Open field—Some comparative data. Annals of the New York Academy of Sciences. 1969;159(A3):831–&. 10.1111/j.1749-6632.1969.tb12982.x 4981885

[92] Archer J. Tests for emotionality in rats and mice—review. Animal Behaviour. 1973;21(MAY):205–35. 10.1016/s0003-3472(73)80065-x 4578750

[93] Ossenkopp KP, Sorenson L, Mazmanian DS. Factor-analysis of Open-field behavior in the rat (*Rattus norvegicus*)–Application of the 3-way Parafac model to a longitudinal data set. Behavioural Processes. 1994;31(2–3):129–44. 10.1016/0376-6357(94)90001-9 . 24924928

[94] Chiszar D, Carter T, Knight L, Simonsen L, Taylor S. Investigatory behavior in Plains garter snake (*Thamnophis radix*) and several additional species. Animal Learning & Behavior. 1976;4(3):273–8. 10.3758/bf03214049

[95] Suchomelová P, Jančúchová-Lásková J, Landová E, Frynta D. Experimental assessment of social interactions in two species of the genus *Teratoscincus* (Gekkota). Behavioural Processes. 2015;120:14–24. 10.1016/j.beproc.2015.08.005 . 26299547

[96] Le Galliard JF, Paquet M, Mugabo M. An experimental test of density-dependent selection on temperament traits of activity, boldness and sociability. Journal of Evolutionary Biology. 2015;28(5):1144–55. 10.1111/jeb.12641 . 25865798

[97] Lopez P, Hawlena D, Polo V, Amo L, Martin J. Sources of individual shy-bold variations in antipredator behaviour of male Iberian rock lizards. Animal Behaviour. 2005;69:1–9. 10.1016/j.anbehav.2004.05.010

[98] Brodie ED. Consistency of individual differences in antipredator behavior and color pattern in the garter snake, *Thamnophis ordinoides*. Animal Behaviour. 1993;45(5):851–61. 10.1006/anbe.1993.1106

[99] Herzog HA, Bowers BB, Burghardt GM. Development of antipredator responses in snakes. V. Species differences in ontogenetic trajectories. Developmental Psychobiology. 1992;25(3):199–211. 10.1002/dev.420250305 . 1618371

[100] Herzog HA, Bowers BB, Burghardt GM. Development of antipredator responses in snakes. IV. Interspecific and intraspecific differences in habituation of defensive behavior. Developmental Psychobiology. 1989;22(5):489–508. 10.1002/dev.420220507 . 2759360

[101] Aloisi AM, Ceccarelli I, Lupo C. Behavioural and hormonal effects of restraint stress and formalin test in male and female rats. Brain Research Bulletin. 1998;47(1):57–62. 10.1016/s0361-9230(98)00063-x . 9766390

[102] Lillywhite HB, Zippel KC, Farrell AP. Resting and maximal heart rates in ectothermic vertebrates. Comparative Biochemistry and Physiology a-Molecular and Integrative Physiology. 1999;124(4):369–82. 10.1016/s1095-6433(99)00129-4 10682235

[103] Carrasco JL, Jover L. Concordance correlation coefficient applied to discrete data. Statistics in Medicine. 2005;24(24):4021–34. 10.1002/sim.2397 . 16320280

[104] Team RDC. R: a language and environment for statistical computing. In: Computing RFfS, editor. Vienna, Austria 2012.

[105] Statsoft S. Statistica. 6.0 ed2001.

[106] Budaev SV. Using Principal Components and Factor Analysis in Animal Behaviour Research: Caveats and Guidelines. Ethology. 2010;116(5):472–80. 10.1111/j.1439-0310.2010.01758.x

[107] Flores D, Tousignant A, Crews D. Incubation temperature affects the behavior of adult leopard geckos (*Eublepharis macularius*). Physiology & Behavior. 1994;55(6):1067–72. 10.1016/0031-9384(94)90389-1 8047573

[108] Luttbeg B, Sih A. Risk, resources and state- dependent adaptive behavioural syndromes. Philosophical Transactions of the Royal Society B-Biological Sciences. 2010;365(1560):3977–90. 10.1098/rstb.2010.0207 PMC2992746 21078650

[109] Dingemanse NJ, Wolf M. Recent models for adaptive personality differences: a review. Philosophical Transactions of the Royal Society B-Biological Sciences. 2010;365(1560):3947–58. 10.1098/rstb.2010.0221 PMC2992752 21078647

[110] Herczeg G, Ab Ghani NI, Merila J. Evolution of stickleback feeding behaviour: genetics of population divergence at different ontogenetic stages. Journal of Evolutionary Biology. 2013;26(5):955–62. 10.1111/jeb.12103 . 23458103

[111] Verbeek MEM, Drent PJ, Wiepkema PR. Consistent individual differences in early exploratory behavior of male Great tits. Animal Behaviour. 1994;48(5):1113–21. 10.1006/anbe.1994.1344

[112] Stowe M, Kotrschal K. Behavioural phenotypes may determine whether social context facilitates or delays novel object exploration in ravens (*Corvus corax*). Journal of Ornithology. 2007;148:S179–S84. 10.1007/s10336-007-0145-1

[113] Herborn KA, Coffey J, Larcombe SD, Alexander L, Arnold KE. Oxidative profile varies with personality in European greenfinches. Journal of Experimental Biology. 2011;214(10):1732–9. 10.1242/jeb.051383 21525320

[114] Montiglio PO, Garant D, Bergeron P, Messier GD, Réale D. Pulsed resources and the coupling between life-history strategies and exploration patterns in eastern chipmunks (*Tamias striatus*). Journal of Animal Ecology. 2014;83(3):720–8. 10.1111/1365-2656.12174 . 24180283

[115] Jones KA, Godin JGJ. Are fast explorers slow reactors? Linking personality type and anti-predator behaviour. Proceedings of the Royal Society B-Biological Sciences. 2010;277(1681):625–32. 10.1098/rspb.2009.1607 PMC2842688 19864291

[116] Verbeek MEM, Boon A, Drent PJ. Exploration, aggressive behavior and dominance in pair-wise confrontations of juvenile male great tits patent 11–12. 1996 9.

[117] van Oers K, Drent PJ, de Goede P, van Noordwijk AJ. Realized heritability and repeatability of risk-taking behaviour in relation to avian personalities. Proceedings of the Royal Society B-Biological Sciences. 2004;271(1534):65–73. 10.1098/rspb.2003.2518 PMC1691563 15002773

[118] Koolhaas JM, Korte SM, De Boer SF, Van Der Vegt BJ, Van Reenen CG, Hopster H, et al Coping styles in animals: current status in behavior and stress-physiology. Neuroscience and Biobehavioral Reviews. 1999;23(7):925–35. 10.1016/s0149-7634(99)00026-3 . 10580307

[119] Koolhaas JM, de Boer SF, Coppens CM, Buwalda B. Neuroendocrinology of coping styles: Towards understanding the biology of individual variation. Frontiers in Neuroendocrinology. 2010;31(3):307–21. 10.1016/j.yfrne.2010.04.001 . 20382177

[120] DuRant SE, Romero LM, Talent LG, Hopkins WA. Effect of exogenous corticosterone on respiration in a reptile. General and Comparative Endocrinology. 2008;156(1):126–33. 10.1016/j.ygcen.2007.12.004 . 18249406

[121] Langkilde T, Shine R. How much stress do researchers inflict on their study animals? A case study using a scincid lizard, *Eulamprus heatwolei*. Journal of Experimental Biology. 2006;209(6):1035–43. 10.1242/jeb.02112 16513929

[122] Carere C, van Oers K. Shy and bold great tits (*Parus major*): body temperature and breath rate in response to handling stress. Physiology & Behavior. 2004;82(5):905–12. 10.1016/j.physbeh.2004.07.009 15451657

[123] Stinner JN, Ely DL Blood pressure during routine activity, stress, and feeding in Black racer snakes (*Coluber constrictor*). American Journal of Physiology. 1993;264(1):R79–R84 8430890 10.1152/ajpregu.1993.264.1.R79

[124] Secor SM, Hicks JW, Bennett AF. Ventilatory and cardiovascular responses of a python (*Python molurus*) to exercise and digestion. Journal of Experimental Biology. 2000;203(16):2447–54 10903159 10.1242/jeb.203.16.2447

[125] Stamps JA, Krishnan VV. Combining Information from Ancestors and Personal Experiences to Predict Individual Differences in Developmental Trajectories. American Naturalist. 2014;184(5):647–57. 10.1086/678116 . 25325748

[126] del Barrio V, Moreno-Rosset C, Lopez-Martinez R, Olmedo M. Anxiety, depression and personality structure. Personality and Individual Differences. 1997;23(2):327–35. 10.1016/s0191-8869(97)00030-5

[127] Gullone E, Moore S. Adolescent risk-taking and the five-factor model of personality. Journal of Adolescence. 2000;23(4):393–407. 10.1006/jado.2000.0327 . 10936013

