# Supplementary material for: Development of behavioural profile in the Northern common boa (Boa imperator): Repeatable independent traits or personality?
Source: PLoS One. 2017 May 24;12(5):e0177911. doi: 10.1371/journal.pone.0177911 (PMC5443515; doi:10.1371/journal.pone.0177911)
Supplement: S1 File — The list of contexts, tests and variables measured in our manuscript. More detailed definitions of these variables are provided. (DOC) [file pone.0177911.s001.doc]

**S1 File. Definitions of Variables**

This supplementary material contains more detailed definitions of the variables analyzed in our paper. The scheduling of the trials can be found in Figure 1 and Table 1.

**Foraging and feeding context**

**Feeding trials**

***Prey acceptance.*** Prey acceptance was calculated as a proportion of successful feeding trials of a snake in a particular feeding block. We considered the feeding trial as successful, if the snake decided to feed; i.e., consumed at least one of the offered prey items. Prey acceptance could vary between 0 (no accepted food in the block) and 1 (all feeding trials were successful). The prey (live or death) was considered as refused the next day, when it was removed from the cage. The values were square-root arcsin transformed prior to the statistical analysis.

***Catch latency of familiar prey.*** We defined catch latency as the time the snake needs to catch the prey successfully. The time was recorded from introducing to the moment the prey was seized with the open mouth. We used two ways of expressing this variable. Firstly, we measured the time in seconds, until the prey was successfully caught (usually majority of the snakes caught the prey within 20 minutes). If the snake refused the prey item, it was given a latency of 10 hours (36 000 sec.), corresponding to the maximal duration of the trial. This precise measurement was also done twice for a familiar prey.

***Index of catch latency.*** During regular feeding trials, we also checked the latency to catch the prey. We scored this latency on the categorial ordinal scale: 1 - less than three seconds, 2 - between three and ten seconds, 3 - between ten seconds and one hour and 4 - more than one hour. We calculated the Index of catch latency as an average score per the feeding block. We used a log-transformed values for the analyses. Defensive attacks without subsequent constriction of the prey were excluded together with trials without any foraging activity.

**Boldness in novel prey context**

**Novel prey test**

Novel prey tests were performed at the age of 534 days of life (live chicken), 745 days (dead spiny mouse *Acomys* sp.), 805 days of life (chicken neck), 812 days of life (dead chicken) and 842 days of life (spiny mouse *Acomys* sp.).

***Catch latency of novel prey.*** We used catch latency as a main variable in novel prey tests, defined as the time the snake needs to catch the new prey successfully (the procedure was similar to the measurement of the Catch latency of familiar prey (see above). We offered novel prey of different kind to the snakes six times with different types of prey. We include the catch latencies measured in five novel prey experiments (with chicken, dead spiny mouse, chicken neck, dead chicken and live spiny mouse offered to the snake for the first time) into the variable named Catch latency novel. We added the catch latency of a mouse pup that was offered to the snake as the first prey ever to the variable Capture success novel. This variable includes latencies to catch different types of live prey (pup, live chicken, live spiny mouse). We coded this variable as binomial: successful (1) / unsuccessful (0) catch during the first 20 minutes. This was due to the binomial distribution of the measured hunting activity of juveniles (those catching prey immediately vs those with very long latencies, e.g., several hours). We also analysed the catch latencies of live prey across all life stages as a continuous variable named Catch latency novel (live prey) including novel chicken and novel spiny mouse, that was log transformed for achieving normal distribution. Similarly, we pooled the experiments measuring the snake’s latency to catch novel dead prey (dead spiny mouse, chicken neck, dead chicken) in a variable named Catch latency novel (dead prey).

**Context of exploration**

**Open field**

***Movement latency and time to leave.*** Some snakes readily moved after being placed into the arena, but after a few seconds, they stopped and stayed inactive for the rest of the trial. This behaviour was measured similarly to those who did not move at all. Thus, we added a measurement of the latency to the first continuous movement of the animals (at least one third of the snake’s body was moving). We named this variable Movement latency. As we only used a virtual arena with no surrounding walls, we also measured the time to leave the arena. Latency of the first movement and the time needed to leave the arena were recorded, up to the limit of 450 seconds. We named the variable Time to leave the area of the arena and we used the log-transformation for the normalisation of the data.

**Activity test**

We recorded the activity of the snake for 24 hours in the home cage and for 20 hours in the novel cage and continually measured the duration of time spent as active and the duration of time spent as passive. The snake was considered to be active if moved continuously, or moved more than one third of the body for longer than 15 seconds. Immobility, even with a part of the body elevated above the ground, was classified as passivity. The variable Percentage of movement was calculated as the proportion of the time spent active during the trial.

**Agonistic behaviour context**

**Reactivity test**

The Reactivity test was repeated three times: first several days after the birth, and then in one and two years of age.

***Movement.*** We recorded the occurrence of any locomotor activity during the Reactivity test, as many of the snakes were immobile as a response to threat. The defensive behaviour was so rare that we excluded it from the analysis.

***Tongue flicking.*** We also counted the number of tongue flicks, which reflect olfactory and chemical exploration.

**Handling test**

***Index of defensive behaviour.*** The long-term monitoring of defensive behaviour refers to an occurrence of any type of the defensive behaviour during any handling trial in a particular block and is calculated similarly as the *Prey acceptance.* When the snake showed any aggressive behaviour in the trial, it was scored as 1, otherwise it was scored as a 0. The Index of defensive behaviour was calculated as a proportion of handling trials scored with 1 per block.

**Restraint test**

***Occurrence of defensive behaviour.*** Hissing, striking and attacking can be clearly attributed to the defensive behaviour. For the purposes of this study, we defined the various defensive traits as follows: striking is characterized by non-directional strikes, in contrast to directed (usually towards hands) attacks accompanied with bites. Defensive attacks differ from the hunting behaviour. They are superficial and very short-time lasting; presumably, the purpose of defensive striking and bites is to startle or warn (“do not approach”) rather than to cause a noticeable injury. We recorded a binary-coded variable (0/1) named the Occurrence of defensive behaviour during the Restraint test.

**Stress response test**

***Heart and breath rate.*** Movements of the snakes’ body enabled us to record breathing of the snakes visually. Heart beat was detected by hand-held ultrasound transducer (Doppler heart rate monitor). This method does not guarantee obtaining the real resting heart rates even in snakes habituated to handling. Despite the possible small deviation from the real resting heart rate, this non-invasive method enabled us to repeat the measurements without any health risk for the animals or interaction with other behavioural tests.

The heart beat and breathing frequency and intervals of the beats and breaths were analysed using the computer program package ACTIVITIES. We checked the records and manually corrected the apparent errors before proceeding to the next analysis.

***Heart and Breath Rate Stress Response.*** We repeated the measurements of heart rate and breath rate in non-stress and stress situation twice. For both variables, measured in non-stress as well as in stress situations, we checked for the repeatability of these measurements within three-minute test (e.g., heart beat in first, second and third minutes of the experiment) in order to detect the dynamic of this reaction. We also calculated the repeatability between the tests for both variables, and non-stress as well as stress situation. Finally, we calculated the variable reflecting individual stress response for both variables and both tests. We used the difference between the lowest (closest to the resting value) and the highest (the strongest response to the stress stimulus) recorded frequency from the test as the measure of the stress response. We took the minimal value from the non-stressful situation (minimal resting value) and the maximal value measured in stress situation for each individual and then we calculated their difference.
